# Pan-African phylogeny of *Mus* (subgenus *Nannomys*) reveals one of the most successful mammal radiations in Africa

**DOI:** 10.1186/s12862-014-0256-2

**Published:** 2014-12-14

**Authors:** Josef Bryja, Ondřej Mikula, Radim Šumbera, Yonas Meheretu, Tatiana Aghová, Leonid A Lavrenchenko, Vladimír Mazoch, Nicholas Oguge, Judith S Mbau, Kiros Welegerima, Nicaise Amundala, Marc Colyn, Herwig Leirs, Erik Verheyen

**Affiliations:** Institute of Vertebrate Biology, Academy of Sciences of the Czech Republic, Brno, Czech Republic; Department of Botany and Zoology, Faculty of Science, Masaryk University, Brno, Czech Republic; Institute of Animal Physiology and Genetics, Academy of Sciences of the Czech Republic, Brno, Czech Republic; Department of Zoology, Faculty of Science, University of South Bohemia, České Budějovice, Czech Republic; Department of Biology, College of Natural and Computational Sciences, Mekelle University, Tigray, Ethiopia; A.N.Severtsov Institute of Ecology and Evolution RAS, Moscow, Russia; Earth Watch Institute, Nairobi, Kenya; College of Agriculture and Veterinary Sciences, University of Nairobi, Nairobi, Kenya; University of Kisangani, Eastern Province, Kisangani, DR Congo; CNRS UMR 6552/53, Université de Rennes 1, Station Biologique, Paimpont, France; Evolutionary Ecology Group, Biology Department, University of Antwerp, Antwerpen, Belgium; Royal Belgian Institute for Natural Sciences, Operational Direction Taxonomy and Phylogeny, Brussels, Belgium; Institute of Vertebrate Biology, Academy of Sciences of the Czech Republic, Research Facility Studenec, Studenec 122, 675 02 Koněšín, Czech Republic

**Keywords:** Biogeography, Tropical Africa, Molecular phylogeny, Pygmy mice, Plio-Pleistocene climatic fluctuations, Divergence timing, Muridae (Murinae), *Mus minutoides*, Phylogeography, DNA barcoding

## Abstract

**Background:**

Rodents of the genus *Mus* represent one of the most valuable biological models for biomedical and evolutionary research. Out of the four currently recognized subgenera, *Nannomys* (African pygmy mice, including the smallest rodents in the world) comprises the only original African lineage. Species of this subgenus became important models for the study of sex determination in mammals and they are also hosts of potentially dangerous pathogens. *Nannomys* ancestors colonized Africa from Asia at the end of Miocene and Eastern Africa should be considered as the place of their first radiation. In sharp contrast with this fact and despite the biological importance of *Nannomys*, the specimens from Eastern Africa were obviously under-represented in previous studies and the phylogenetic and distributional patterns were thus incomplete.

**Results:**

We performed comprehensive genetic analysis of 657 individuals of *Nannomys* collected at approximately 300 localities across the whole sub-Saharan Africa. Phylogenetic reconstructions based on mitochondrial (*CYTB*) and nuclear (*IRBP*) genes identified five species groups and three monotypic ancestral lineages. We provide evidence for important cryptic diversity and we defined and mapped the distribution of 27 molecular operational taxonomic units (MOTUs) that may correspond to presumable species. Biogeographical reconstructions based on data spanning all of Africa modified the previous evolutionary scenarios. First divergences occurred in Eastern African mountains soon after the colonization of the continent and the remnants of these old divergences still occur there, represented by long basal branches of *M.* (previously *Muriculus*) *imberbis* and two undescribed species from Ethiopia and Malawi. The radiation in drier lowland habitats associated with the decrease of body size is much younger, occurred mainly in a single lineage (called the minutoides group, and especially within the species *M. minutoides*), and was probably linked to aridification and climatic fluctuations in middle Pliocene/Pleistocene.

**Conclusions:**

We discovered very high cryptic diversity in African pygmy mice making the genus *Mus* one of the richest genera of African mammals. Our taxon sampling allowed reliable phylogenetic and biogeographic reconstructions that (together with detailed distributional data of individual MOTUs) provide a solid basis for further evolutionary, ecological and epidemiological studies of this important group of rodents.

**Electronic supplementary material:**

The online version of this article (doi:10.1186/s12862-014-0256-2) contains supplementary material, which is available to authorized users.

## Background

One of the main challenges of current nature conservation is the accelerating loss of biodiversity. Even if this problem is generally recognized, there are several difficulties in quantifying the loss of biodiversity at the level of species. For example, there is a lack of traditional taxonomic specialists for particular groups of organisms and the real amount of biodiversity is therefore unknown [1]. This is especially true for some tropical areas, where the overall biodiversity level is the highest and its loss is the most intensive. Another problem for practical biodiversity conservation is the delimitation of species (e.g. [2] vs. [3]). Traditional concepts of typological or biological species are not universally applicable and with accumulating knowledge in evolutionary biology it is increasingly difficult to define generally what a species is. Genetic approaches, like DNA barcoding, are now routinely used to overcome some of these problems. They provide a cheap and easily applicable approach for discovering the taxa worth future taxonomical research and areas with high phylogenetic diversity with special conservation concern (e.g. [4]). For example, 175 new extant taxa of mammals were described from African mainland, Madagascar and all surrounding islands between 1988–2008 [5], and in the majority, the first consideration for taxonomic delimitation was motivated by the use of genetic data.

Rodents of the genus *Mus* represent one of the most valuable biological models for biomedical and evolutionary research [6]. Out of the four currently recognized subgenera, i.e. *Mus*, *Coelomys*, *Pyromys* and *Nannomys*, the latter comprises the African pygmy mice [7]. These are small rodents (4–12 g in most taxa, but see [8]), endemic to the sub-Saharan Africa. The phylogenetic relationships, species diversity, ecology and chromosomal evolution of *Nannomys* were recently reviewed [9]. They represent the most diverse lineage of the genus, with currently about 18 species recognized [9,10], comprising almost half of the described *Mus* species [10]. While predominantly savannah dwellers [11], several species have also been trapped in forest, agricultural fields and rural areas [12-14].

Mainly due to their extensive chromosomal diversity coupled with highly conserved morphology, African pygmy mice have attracted the attention of evolutionary scientists [9,11,15-17]. They exhibit chromosomal features that are rarely recorded in other taxa, e.g. the greatest diversity of sex-autosome translocations reported so far in any mammalian lineage (e.g. [18]). Thus *Nannomys* became an important biological model for the study of processes of chromosomal speciation and mechanisms of sex determination in mammals [19]. Recent studies have also shown that African pygmy mice are important hosts of arena viruses [20-23], making them a target group for epidemiological surveys.

Increasing numbers of molecular genetic data provide evidence for high cryptic diversity in *Nannomys* and it is highly probable that further integrative taxonomy research will reveal new undescribed species [11,14]. Furthermore, the inclusion of poorly known African *Mus*-related rodents in molecular phylogenetic datasets may provide surprising results changing the current view on the evolutionary scenarios of *Nannomys*. For example, the Ethiopian endemic genus *Muriculus* was recently recognized to be an internal lineage of *Mus* [8].

**Figure 1:**
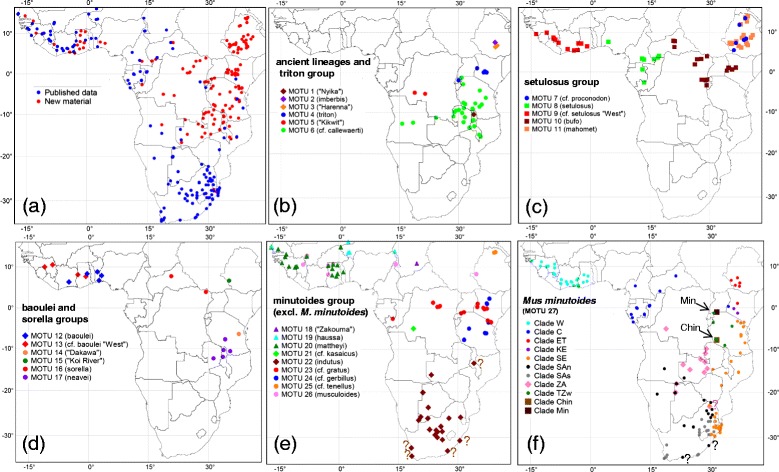
**Distribution of genotyped specimens and individual MOTUs. (a)** Distribution of analyzed material of *Nannomys* in Africa. Blue dots indicate the geographical position of published sequences (downloaded from GenBank), red dots show the localities of newly sequenced individuals. Geographical distribution of **(b)** MOTUs of the triton group (circles) and three ancient monotypic lineages (rhombuses); **(c)** MOTUs of the setulosus group; **(d)** MOTUs of the baoulei (rhombuses) and the sorella (circles) groups; **(e)** MOTUs of the minutoides group (except *M. minutoides*); **(f)** phylogeographic structure of MOTU 27, i.e. *M. minutoides*. In Figures 1e identical symbol shapes represent monophyletic groups. In Figure 1f the clade abbreviations correspond to Figure 4. Question marks indicate doubtful records based on genotyping of old museum material (see [64]). For more information on analysed material see Additional file 1.

The genus *Mus* diverged in Asia approximately 6.7 to 7.8 Mya and shortly after this time the ancestor(s) of *Nannomys* colonized Africa through the Arabian Peninsula and Miocene land bridges [9]. The oldest fossils of *Mus* in Africa are reported from Tugen Hills (Kenya) about 4.5 Mya [24]. The highly heterogeneous environment of Eastern Africa can thus be considered as the place of first diversification of African *Mus* in Early Pliocene, followed by a radiation caused by climatic oscillations and habitat modification [9,11]. In this context it is important to note that genetic data used so far for molecular phylogenetic inference of the African pygmy mice are strongly biased geographically in favour of material collected from savannahs in the western and southern part of the continent, while specimens from Eastern Africa (including those from mountains and forests) are clearly under-represented (Figure 1a).

More thorough geographical sampling is necessary for obtaining the correct biogeographical scenario of *Nannomys* evolution. Only a comprehensive and reliable phylogenetic hypothesis can lead to meaningful inferences on the evolution of sex-determination or virus-host co-evolution. In this study, we provide the so far most comprehensive geographic sampling of genetically characterized African pygmy mice composed of 657 *Nannomys* individuals from most parts of sub-Saharan Africa. First, we use this pan-African dataset for the reconstruction of phylogenetic relationships within *Nannomys* lineage. Second, using the combination of species delimitation methods, we aim to estimate the presumable species richness of *Nannomys*, highlighting groups and geographical regions necessitating further taxonomical research. Finally, the dating of divergences and biogeographical reconstructions allow us to modify previous scenarios that were suggested to explain the *Nannomys* radiation in Africa.

## Methods

### Sampling

New genetic data were produced for 395 individuals of subgenus *Nannomys* sampled in sub-Saharan Africa by the authors and their collaborators. All fieldwork complied with legal regulations in particular African countries and sampling was in accordance with local legislation (see more details on wildlife authorities that permitted the research in Acknowledgements). Each individual was identified to the genus by the external features and the tissue sample (tail, toe, spleen, etc.) was stored in 96% ethanol until DNA extraction. GPS coordinates of each locality were recorded. New data were supplemented with 262 published records of genotyped and georeferenced *Nannomys*, i.e. partial or complete sequences of mitochondrial gene for cytochrome *b* (*CYTB*) were downloaded from GenBank. Geographical coordinates of published data were either retracted from original publications (if available) or approximately estimated from Google maps. In total, the analysed dataset includes genetic information of 657 individuals from approximately 300 localities in 30 African countries (Figure 1a). For more details on analysed individuals see Additional file 1.

### DNA sequencing of *CYTB* and *IRBP*

DNA was extracted using the DNeasy Blood & Tissue Kit (Qiagen). The complete *CYTB* gene was amplified by polymerase chain reaction (PCR) using primers L14723 and H15915 [25]. PCR mix contained 3 μl of genomic DNA, 0.5 units of Taq polymerase (Fermentas), final concentrations of 3 mM MgCl_2_, 1 x Taq buffer with (NH_4_)_2_SO_4_ (Fermentas), 0.2 mM of each dNTPs, 0.2 μM of each primers and ddH_2_O to final volume of 30 μl. The thermal profile of the PCR started with an initial denaturation at 94°C for 3 min, followed by 35 cycles composed of 30 s of denaturation at 94°C, 30 s of annealing at 50°C, and 3 min of extension at 72°C and PCR was finished by a final extension at 72°C for 10 min. The part of nuclear gene encoding the Interphotoreceptor Binding Protein (*IRBP*) was amplified in selected individuals (from each main clade identified previously by *CYTB* marker) by the primers IRBP1531 and IRBP217 [26]. PCR conditions were the same as above, except the final concentration of MgCl_2_ (2 mM). The thermal profile of the PCR started with an initial denaturation of one step at 94°C for 3 min, followed by 30 cycles of 60 s at 94°C, 60 s at 55°C, 2 min at 72°C and finished by a final extension at 72°C for 10 min. The PCR products were purified with Calf Intestine Alkaline Phosphatase (ThermoScientific) and Exonuclease I (ThermoScientific) and sequenced along both strands commercially in Macrogen Europe using the same primers as for the PCR. Both genetic markers have been previously used to successfully resolve systematic relationships in a wide range of related murid rodents (e.g. [7,25].

Genetic data from fresh material were complemented by museum samples (mostly dry skins) from the Royal Museum for Central Africa (Tervuren, Belgium), Muséum National d'Histoire Naturelle (Paris, France), American Museum of Natural History (New York, USA) and Hungarian Natural History Museum (Budapest, Hungary) (for more details see Additional file 1). Museum samples comprised only minor part of analysed material and we used especially those from geographical areas difficult to be accessed today (e.g. Central African Republic, eastern Democratic Republic of Congo) and the type material of *Mus bufo*. All museum samples were handled in a specialized laboratory of Institute of Vertebrate Biology ASCR in Studenec, designed for work with rare DNA to prevent contamination by samples with high quantity of DNA or PCR products. DNA was extracted using the JETQUICK Tissue DNA Spin Kit (Genomed). PCR amplification and pyrosequencing on GS Junior were performed according to mini-barcode protocol described by [27]. The main advantage of this approach in analysis of museum samples is that it allows for separating individual sequences in samples contaminated by distantly related organisms (e.g. contamination by human DNA), which is not possible through the Sanger sequencing.

### Phylogenetic reconstructions

Sequences of *CYTB* and *IRBP* were edited and aligned in SeqScape v2.5 (Applied Biosystems), producing a final alignment of 1140 and 1276 bp, respectively. The Findmodel web application (http://www.hiv.lanl.gov/content/sequence/findmodel/findmodel.html) was used to identify the most appropriate substitution model for each gene. The Akaike information criterion (AIC), compared among 12 substitution models, revealed that the model best fitting the ingroup data was the General time reversible model with a gamma-distributed rate variation across sites (GTR + G) for both *CYTB* and *IRBP*. As outgroups, we used sequences of four species from other subgenera of the genus *Mus*, i.e. *M. platythrix* (*CYTB* GenBank Acc. code AJ698880, *IRBP* GenBank Acc. code AJ698895), *M. pahari* (AY057814, AJ698893), *M. caroli* (AB033698, AJ698885) and *M. musculus* (V00711, AF126968); two sister lineages of the genus *Mus* within subfamily Murinae, i.e. *Apodemus flavicollis* (AB032853, AB032860) and *Ratus norvegicus* (V01556, AJ429134); and one species from the subfamily Acomyinae, *Acomys cahirinus* (AJ233953, AJ698898) see also [7,9,11].

Phylogenetic relationships within *Nannomys* were inferred by maximum likelihood (ML) and Bayesian (BI) approaches. ML analysis was performed using RAxML 8.0 [28]. The GTR + G model (option -m GTRGAMMA) was selected for the six partitions, i.e. 1140 bp of *CYTB*, 1276 bp of *IRBP*, and both genes were partitioned also by the position of nucleotides in the codons (option -q). The robustness of the nodes was evaluated by the default bootstrap procedure with 1,000 replications (option -# 1000). Bayesian analysis of evolutionary relationships was performed by Markov chain Monte Carlo (MCMC) method in MrBayes v. 3.2.1 [29]. Three heated and one cold chain were employed in all analyses, and runs were initiated from random trees. Two independent runs were conducted with 5 million generations per run; and trees and parameters were sampled every 1,000 generations. Convergence was checked using TRACER v1.5 [30]. For each run, the first 10% of sampled trees were discarded as burn-in. Bayesian posterior probabilities were used to assess branch support of the Bayesian tree.

The most widespread *Nannomys* species (= MOTU, see below) is *M. minutoides*. For this species we performed more detailed analysis of intraspecific genetic variability. We selected 131 sequences belonging to this clade and trimmed the final alignment to the length of 741 bp. Haplotypes were generated using DNaSP software [31] and a median-joining network of haplotypes was produced in the software Network 4.6.1.2 (downloaded on 10.2.2014 from http://www.fluxus-engineering.com/sharenet.htm).

### Delimitation of MOTUs

We estimated the possible number of putative species (called here molecular operational taxonomic units, MOTUs, until the thorough taxonomic evidence will be provided) of *Nannomys* in the sampled dataset by using two types of divergence thresholds and the *CYTB* dataset. The first was the time threshold estimated by the Generalized Mixed Yule Coalescent (GMYC) model [32] which describes single-locus branching pattern as a succession of speciation events replaced at a fixed threshold time by a succession of intraspecific coalescent events. The two stages are modelled by Yule process and neutral coalescent, respectively, which allows finding maximum likelihood estimate of the threshold time and evaluating statistical support for the delimited species [33,34]. In this framework reliably delimited species are those whose basal internal split occurred well after the speciation-coalescence threshold and which diverged from sister species well before it. We therefore calculated two kinds of support: (1) for each intra-specific basal split we calculated relative likelihood that it represents coalescence rather than speciation event by summing up Akaike weights of all threshold times older or equal to its age; (2) for each inter-specific split we calculated relative likelihood that it represents speciation as a sum of Akaike weights of threshold times younger to it. The ultrametric tree required by GMYC was produced by BEAST 1.8.0 [35] with uncorrelated lognormal distribution of substitution rates and lognormal priors for node ages mimicking posteriors from the divergence dating (see below). We used the Yule prior assuming no intra-specific divergences (alternative analyses with a coalescent prior assuming no speciation events lead to almost identical results of GMYC analyses; not shown). The topology was constrained to match the branching order of main lineages observed in the maximum likelihood phylogeny. The GMYC analysis was performed using the R package ‘splits’ (http://r-forge.r-project.org/R/?group_id=333).

The second threshold was based on sequence divergence, taken as a proxy for the amount of genetic difference among distinct gene pools. We therefore analyzed the distribution of Kimura-2 parameter (K2P) corrected genetic distances on *CYTB* among GMYC-delimited species (calculated in Mega 5.05; [36]) and merged the lineages with less than 7.3% genetic distance, i.e. the mean value between sister species of rodents [37]. The resulting groups were designated as molecular operational taxonomic units (MOTUs) and provisional names were assigned to them. It is important to note that the aim of our MOTUs delimitation approach is not to change the current taxonomy, but to highlight the taxa and geographical areas worthy of further taxonomic study, including morphological, ecological and more detailed genetic approaches.

### Divergence dating

Time to the most recent common ancestors (TMRCA) of clades identified by phylogenetic analyses was estimated using a relaxed clock model with substitution rates drawn from an uncorrelated lognormal distribution in BEAST 1.8.0 [35] and three fossil-based calibration points: origin of extant *Mus*, origin of extant *Apodemus* and the *Arvicanthis*/*Otomys* lineage split. To avoid disproportionate impact of *Nannomys* we fitted the evolutionary model to 63 concatenated *CYTB* and *IRBP* sequences representing main lineages of *Nannomys* and correspondingly deep divergences across the tribes Apodemini, Arvicanthini, Malacomyini, Murini, Otomyini and Praomyini (sensu [38]). The data set is reported in detail in the Additional file 2.

Following [39] we used lognormal calibration densities with zero means whose 5% and 95% quantiles were specified by appropriately chosen standard deviations and offsets and corresponded to the fossil derived minimum and maximum ages. In particular the parameters (standard deviation, offset, 5% and 95% quantile) were: (1) 0.74, 7.00, 7.30 and 10.38 for *Mus*, based on the earliest fossil *Mus* and a member of *Progonomys* considered belonging to *Mus* stem lineage [40]; (2) 0.54, 4.89, 5.30 and 7.30 for *Apodemus* corresponding to 95% confidence interval of first appearance as reported by [39], although we applied it to the basal split of extant species rather than to the origin of their stem lineage; (3) 0.80, 5.81, 6.08 and 9.54 for *Arvicanthis*/*Otomys* split which was derived from the earliest records of *Otomys* (ca. 5 Mya; [41]) and arvicanthine genera *Aethomys*, *Arvicanthis* and *Lemniscomys* (6.08–6.12 Mya; [42]) and the next relevant sample where these and related genera (except for a tentative *Aethomys*) are absent (9.50-10.50 Mya; [43]). Based on the previous studies [38,39,44] we constrained the topology to include a basal split between Arvicanthini+Otomyini and the rest of the species.

The MCMC simulations were run twice with 25 million iterations, with genealogies and model parameters sampled every 1000 iterations. Trees were linked, models and clocks were unlinked for two markers. Convergence was checked using TRACER v1.5 [30], both runs being combined in LOGCOMBINER 1.7.1 [35] and the maximum clade credibility tree calculated by TREEANNOTATOR 1.7.1 [35], following the removal of 10% burn-in.

### Biogeographical analysis

Ancestral habitat types were inferred by the Bayesian analysis of discrete traits [45]. It models discrete states of a trait at the end of each branch as a result of a continuous time Markov chain with infinitesimal transition rates determined by an overall transition rate, pair-wise transition probabilities and a base frequency of the states. Following the current implementation in BEAST 1.8.0 we used strict clock time-irreversible model so the overall transition rate was assumed uniform across the tree and transition probabilities were allowed to differ in the opposite directions. Using the distribution data, we coded the 27 MOTUs as inhabiting either (i) tropical forests in the Congo Basin, Central and Western Africa; (ii) mountains in Eastern Africa (various habitats), or (iii) savannah habitats in sub-Saharan Africa. Some MOTUs can inhabit more habitat types (e.g. MOTU 27, *M. minutoides*). The analysis in BEAST does not allow more variants of the tip trait, so we assigned the trait (habitat) that is the most widespread in a particular MOTU (e.g. savannah in *M. minutoides*). The topology was fixed to match relationships between MOTUs on the ML tree and branch lengths were time-calibrated as in the ultrametric tree for GMYC.

Alternatively, we identified ancestral habitat types and rough geographic ranges by using the maximum likelihood approach implemented in the software Lagrange [46,47]. The implemented model estimates geographic range evolution using a phylogenetic tree with branch lengths scaled to time, geographic (habitat) areas for all tips, and an adjacency matrix of plausibly connected areas. We used the same tree and distribution data as in the BEAST analysis described above. We allowed the connection between all three habitats with equal probabilities of each transition. The maximum number of ancestral ranges was set to two. The resulting reconstructions returned all models within two likelihood units of the best model, which we summarized for each daughter branch and plotted in the form of pie-charts along the tree in R [48].

## Results

### Overview of collected data

For the phylogenetic analysis we retained 179 *CYTB* sequences at least 700 bp (133 new sequences and 46 sequences from GenBank) representing as complete a geographical distribution of each clade as possible (Additional file 1). The remaining 478 sequences (usually shorter and/or from the same or close neighbouring localities), including 16 sequences obtained by 454 pyrosequencing of old museum samples, were unambiguously assigned to particular MOTU by neighbour-joining analysis in MEGA 5.05 (bootstrap values higher than 95%) and these data were used for mapping the geographical distribution of phylogenetic clades.

We also selected 1–2 individuals from each of the main significantly supported *CYTB* clades (if the tissues were available) and sequenced them at *IRBP* gene. The final phylogenetic analyses included 42 sequences of *IRBP* (32 new sequences and 10 sequences from GenBank; see Additional file 1) from all main species groups except the baoulei group (see below). ML analyses were performed separately for both genes, and because the topology of trees was very similar (although the resolution of *IRBP* was much lower; Additional file 3), we finally performed both ML and BI reconstructions only using a concatenated *CYTB* and *IRBP* dataset produced in SEAVIEW [49].

### Phylogeny of African *Nannomys*

**Figure 2:**
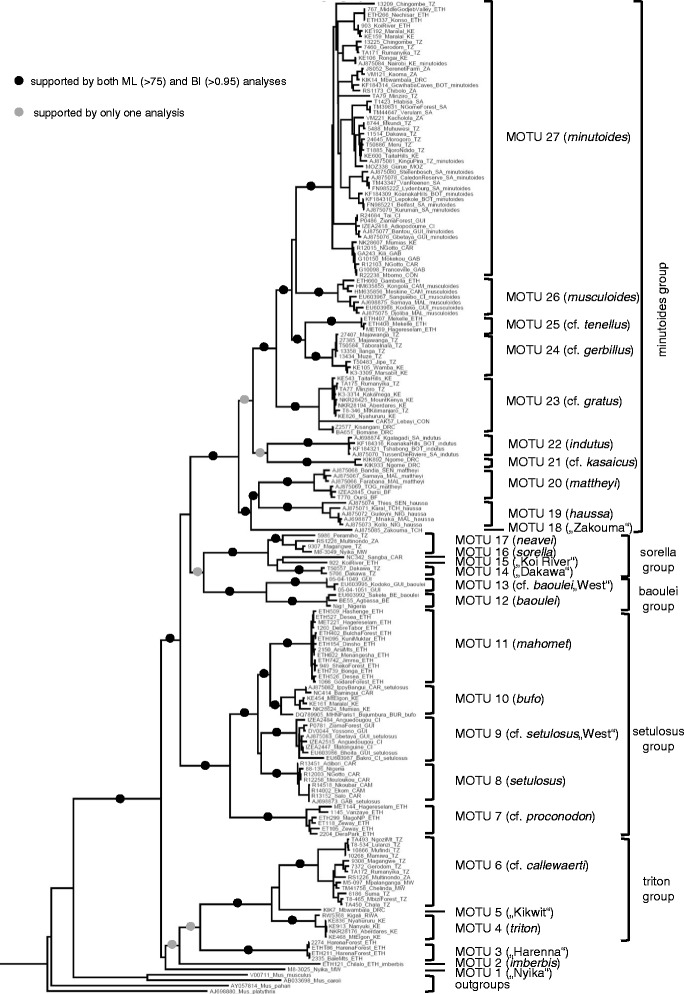
**Inferred phylogenetic relationships within** ***Nannomys*** **.** Maximum likelihood phylogenetic tree of *Nannomys* is based on the combined dataset of mitochondrial (*CYTB*) and nuclear (*IRBP*) genes. Black circles indicate the support by both ML (bootstrap values > 75%) and BI (posterior probabilities > 0.95) analyses; grey circles indicate nodes supported by only one analysis. MOTUs were identified by the combination of GMYC approach and distribution of genetic distances on *CYTB*. Only outgroups from the genus *Mus* are shown. GenBank accession numbers correspond to *CYTB* sequences, for *IRBP* numbers see Additional file 1. Abbreviations of countries: BE: Benin, BF: Burkina Faso, BOT: Botswana, BUR: Burundi, CAM: Cameroon, CAR: Central African Republic, CI: Côte d’Ivoire, CON: Congo, DRC: Democratic Republic of Congo, ETH: Ethiopia, GAB: Gabon, GUI: Guinea, KE: Kenya, MAL: Mali, MOZ: Mozambique, MW: Malawi, NIG: Niger, RWA: Rwanda, SA: South Africa, SEN: Senegal, TOG: Togo, TCH: Tchad, TZ: Tanzania, ZA: Zambia.

Phylogenetic trees based on the concatenated dataset were well resolved and with very similar topology of 179 ingroup sequences in both ML and BI analyses (Figure 2). Subgenus *Nannomys* (including “*Muriculus*” *imberbis*; see [8]) was strongly supported. There are three long branches representing three ancient mountainous species with unresolved relationships to other groups (*M*. sp. “Nyika” = MOTU 1, *M. imberbis* = MOTU 2, and *M*. sp. “Harenna” = MOTU 3) and five well supported species groups. We call them hereafter triton, setulosus, baoulei, sorella, and minutoides groups, based on the previous use of these names, representing the best known species within particular clades. Each group contains several distinct lineages that may represent separate species; the most diversified is the minutoides group. The relationships among species groups are not well resolved, but in most topologies the triton group is non-significantly clustered with three ancient species, while all other species groups cluster together. Within the latter, the setulosus group separates the first, and the baoulei group is the sister of the sorella group (Figure 2).

### Number of potential species and their distribution

**Figure 3:**
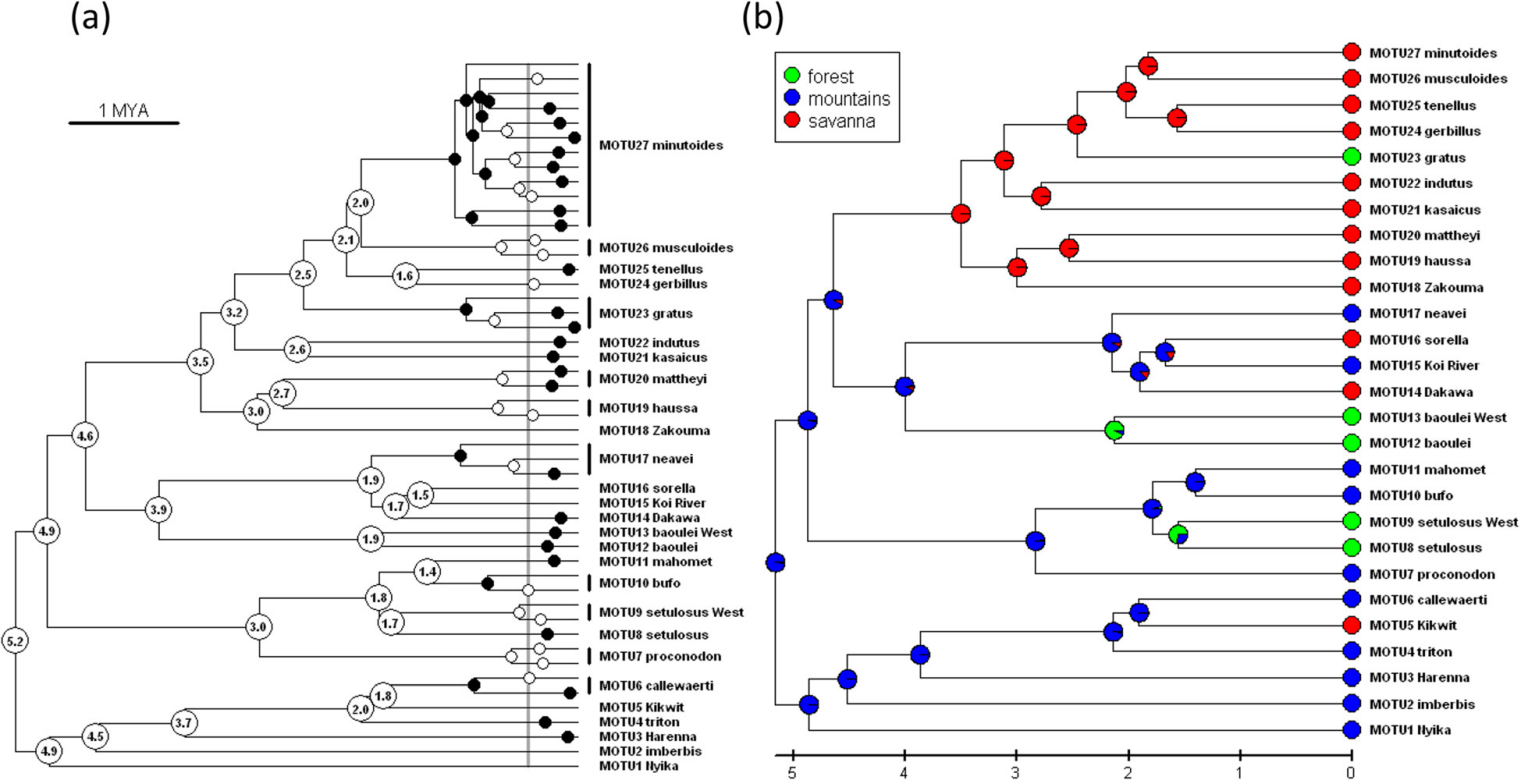
**Reconstruction of divergence dates and ancestral distributions of MOTUs. (a)** Phylogenetic relationships among 49 GMYC-species and definition of 27 MOTUs. The vertical line indicates the threshold where the speciation processes are replaced by coalescence. Black circles indicate strong support (>95%) for either speciation (left of the threshold) or intraspecific coalescence (right of the threshold). White circles indicate weak support (<95%) for these processes. The dating of divergences within *Nannomys* was assessed by BEAST using the previously estimated divergence times (see Additional file 2) as priors for calibration of relaxed molecular clock. **(b)** Reconstruction of ancestral distribution areas (blue – mountains in Eastern Africa, green – tropical forests of central and western Africa; red – open savannah-like habitats surrounding forests and mountains in sub-Saharan Africa. The different colours on pie charts indicate the probability of a particular state of the trait for each node. The analysis of ancestral traits was performed in BEAST (see text for more details).

The application of the GMYC model provided the delimitation of 49 maximum likelihood entities (hereafter GMYC-species; 95% CI = 42–62 entities) based on the ML estimate of speciation-coalescence threshold at 0.46 (0.27–0.86) Mya. Figure 3a depicts support for the “intraspecific” basal splits as coalescences as well as support for “interspecific” splits as speciation events. In both cases white circles indicate support < 0.95 and black circles > 0.95. Low “intraspecific” support suggests there may be more species present, whereas low “interspecific” support suggests the two sister clades may be in fact conspecific populations. Where two neighbouring “interspecific” and “intraspecific” supports are low, the speciation-coalescence transition is blurred.

K2P distances among the GMYC-species (3.16-20.77%) were not overlapping with “intraspecific” distances (0.12-2,38%) (Additional file 4). The detailed analysis of geographical distribution of GMYC-species showed that many sister groups among them are parapatric, i.e. most probably representing the results of allopatric differentiation and secondary contacts. For example, in the clade corresponding to *M. minutoides* in previous studies (e.g. [9]), the GMYC method delimited 12 GMYC-species with prevailing parapatric distribution pattern and with “interspecific” K2P distances 3.27-6.96%. Using the threshold value of 7.3%, we grouped these lineages and considered them as phylogeographical differentiation within the single species *M. minutoides* (see Figure 1f for the distribution of phylogeographical lineages that roughly correspond to “species” identified by GMYC method). Using this combined approach (i.e. analysis of geographic distribution of GMYC-species and threshold of K2P distances), we reduced 49 GMYC-species to 27 highly supported molecular operational taxonomic units (MOTUs, Figure 3a), which are further discussed below. Genetic distances among 27 MOTUs were always significantly higher and did not overlap with those within MOTUs (Additional file 4).

There were 17 MOTUs that exactly matched a single GMYC-species, 11 of them represented by more than one sequence. 7 MOTUs comprised two GMYC-species, 2 MOTUs were composed of three GMYC-species and a single MOTU, MOTU 27 = *M. minutoides*, comprised 12 GMYC-species (Figure 3a). In 12 cases, however, there was strong support for the presence of multiple species within a single MOTU (marked by black circles left of the GMYC threshold in Figure 3a).

Below we follow the nomenclature of [10] that recognizes 18 valid species. Possible names for newly recognized MOTUs are discussed in the text.

1. Ancient mountain lineages (Figure 1b):

A tri-phyletic group with very restricted distribution ranges. They are known from only a few individuals captured in the highest East African mountains. They were not included in previous phylogenetic studies of *Nannomys* and on the phylogenetic tree they form very long branches, in most topologies they are related to the triton group, but not always with significant nodal support.

(MOTU 1) *Mus* sp. “Nyika”

It is a very distinct ancient lineage of *Nannomys*, known from a single, relatively large individual (14 g), captured in the high plateau of Nyika Mts. in Malawi (cca 2100 m a.s.l.). Albeit partially broken, the cranium of this specimen clearly shows features that are typical for insectivorous rodents, namely proodont (forward oriented) incisors and slender mandibles. This lineage is sympatric with MOTU 17 (*M. neavei*) and even syntopic with MOTU 6 (*M*. cf. *callewaerti*).

(MOTU 2) *Mus imberbis* Rüppell, 1842

It is an easily distinguished taxon, very large (sequenced individual weighted 25 g) and with a black dorsal stripe. It has been considered as a separate genus *Muriculus*, but genetic analysis of a recently captured individual clearly shows that it is an internal lineage of *Mus* [8]. It is an endemic species of the high plateaux of Ethiopia, known only from a few of individuals (reviewed in [8]).

(MOTU 3) *Mus* sp. “Harenna”

It is a large species (cca 16 g), very probably endemic to the moist Harenna forest in the Bale Mts. in Ethiopia, a region with very pronounced endemicity [50,51]. Based on morphometry this taxon was previously reported as *M. triton* [50] and in most topologies it is also the sister taxon to the triton group. Genetically it is a very distinct lineage (13.5-14.4% K2P distance to taxa of the triton group) with a remarkably different karyotype than *M. triton* [52]. Earlier studies have already suggested that this taxon represents a valid species [51]. It can be sympatric with *M. mahomet*, but differs in habitat preferences; *M.* sp. “Harenna” lives mostly in the forests, while *M. mahomet* inhabits more open grassy habitats [[53]; L. Lavrenchenko, pers. obs.].

(2) The triton group (Figure 1b):

It is the group of MOTUs of relatively large body size, distributed mostly south of the equator (largely parapatric with the setulosus group - see Figure 1b vs. 1c). Genetic data suggest important cryptic variability (K2P distance among three MOTUs = 8.80-11.05%). Only nominotypical MOTU has a clear valid name, remaining lineages require further taxonomic studies.

(MOTU 4) *Mus triton* (Thomas, 1909)

This species was described from Mt. Elgon in Kenya and we provide the sequence from the type locality. It is distributed in the Kenyan highlands and northern part of Albertine rift. The same species probably occurs in southern Sudan also (described as *M. imatongensis*) [54]), but this should be confirmed by barcoding Sudanese specimens.

(MOTU 5) *Mus* sp. “Kikwit”

This distinct genetic lineage within the triton group was detected in two localities in south-western Democratic Republic of Congo (DRC). It may represent a new species, but more material and analyses are necessary to substantiate this claim. This MOTU supports important biogeographical distinctiveness of the Kikwit region in DRC (see also MOTU 21 from the minutoides group). The type locality of *Mus callewaerti* (Thomas, 1925) (Kananga, Kasaï occidental, DRC) is relatively near, so it is possible that they are conspecific, but a comparison with the type material will be necessary before a final conclusion can be reached (see also MOTU 6).

(MOTU 6) *Mus* cf. *callewaerti*

This taxon forms a well-supported separate lineage within the *triton* group. Its distribution range comprises a fairly important area situated between the Tanzanian Eastern Arc Mountains, through Southern Rift Mountains and northern Zambia till the Angolan highlands. In miombo woodlands of north-western Tanzania, it may have overlapping distribution ranges with *M. triton*, but no locality with sympatric occurrence was found in our study. The Angolan specimens were recently reported as *M. callewaerti* (Thomas, 1925) [14]. It is therefore possible that the whole clade should belong to *M. callewaerti*, but a comparison with type material will be necessary. The taxon prefers the miombo woodland or montane forest edges. There is important genetic variability within this taxon, with animals from Eastern Arc Mountains forming a distinct clade supported as a separate GMYC-species (Figure 3a).

(3) The setulosus group (Figure 1c):

We recognized five MOTUs within this highly supported monophyletic lineage. It includes relatively large-bodied species, with distribution ranges mostly north of the equator, i.e. largely parapatric with the triton group. Two of these MOTUs were only recorded in Ethiopia.

(MOTU 7) *Mus* cf. *proconodon*

It represents a lineage probably endemic to Ethiopia, where it mainly occurs in lowlands of the Rift Valley. We suggest assigning this MOTU to the species *M. proconodon* Rhoads, 1896, i.e. the Ethiopian taxon that was synonymised with *M. setulosus* [10] even if genetically it represents the most distinct lineage of the whole *setulosus* group.

(MOTU 8) *Mus setulosus* Peters, 1876

This highly supported MOTU from western-central Africa (north-west of the Congo River) represents the true *M. setulosus* (type locality is Victoria, Cameroon). The western border of its distribution likely lies in the dry region of the Dahomey gap. In the north-east (i.e. southern Central African Republic (CAR)), it is probably in contact with *M. bufo* (MOTU 10), and it is worthy of further study to analyse the possible contact zone and reproductive barriers between these two taxa in CAR.

(MOTU 9) *Mus* cf. *setulosus* “West”

MOTUs 8–11 form a monophyletic group of four strongly supported lineages with roughly parapatric distribution (Figure 1c). Two of them (MOTUs 8 and 9) have been previously named *M. setulosus* (e.g. [9]). MOTU 8 is distributed in central African forests, while MOTU 9 in western Africa (west of the Dahomey gap). MOTUs 10 and 11 represent valid species *M. bufo* (Thomas, 1906) and *M. mahomet* Rhoads, 1896, respectively. The topology and genetic distances (K2P distance = 8.1%) suggest that MOTUs 8 and 9 should be given different names. Because *M. setulosus* was described from Cameroon (i.e. distribution area of MOTU 8), we suggest that the West African populations of *M.* cf. *setulosus*, i.e. MOTU 9, may represent a separate new species, but this claim needs to be substantiated by further taxonomic work.

(MOTU 10) *Mus bufo* (Thomas, 1906)

The species was described from Ruwenzori Mts. in Uganda and it was considered endemic to the Albertine Rift. There are few sequences identified as *M. bufo* in GenBank. The first (Acc. no. DQ789905) from Bujumbura in Burundi was reported by [9] as an incorrectly assigned species. Recently, new sequences of *M. bufo* from Kahuzi-Biega (DRC) were published [14] and all clearly cluster with the new sequences from CAR, DRC and Kenya reported in our study. Furthermore, we obtained a short sequence from the paratype of *M. bufo* from DRC (locality Idjwi) that also grouped with this clade. Although the morphological comparison with additional type material is necessary, we suggest that *M. bufo* has a much larger distribution range than previously assumed. This taxon may also involve additional populations of the setulosus group from Eastern Africa, especially those assigned to *M. emesi* Heller, 1911 (described from Uganda; morphologically similar to *M. mahomet*, with which it was synonymised [10]), and *M. pasha* Thomas, 1910 (East-African taxon that was synonymized first with *M. proconodon* and later on with *M. setulosus* [10]).

(MOTU 11) *Mus mahomet* Rhoads, 1896

It is an abundant species with a distribution range restricted to the Ethiopian Plateau. We provide the first sequences of this taxon, confirming its position within the setulosus group as a strongly supported monophyletic lineage. We therefore support the view of [55], who considered *M. mahomet* as an Ethiopian endemic, contrary to previous opinions merging it with Kenyan and Ugandan populations (i.e. most probably with MOTU 10, which is significantly supported sister group to *M. mahomet*; Figure 2).

(4) The baoulei group (Figure 1d):

This is a West African clade, until now known as a single species, but with very pronounced divergences between two subclades (mean K2P distance on *CYTB* = 9.46%) that have partially overlapping distribution ranges in Ghana and Ivory Coast. Only very limited genetic data are available, because the species of the baoulei group are probably rare or difficult to capture [12,13,23]. The species of this group occur in the forest-savannah ecotone and are generally larger than other West African species (except *M. setulosus*) [12]. The baoulei group is a sister lineage to the *sorella* group (Figure 2), which is also reflected in morphology [56].

(MOTU 12) *Mus baoulei* (Vermeiren & Verheyen, 1980)

The species *M. baoulei* was described from Lamto in the Ivory Coast [56]. Two individuals sequenced from the type locality [12] belong to the genetic clade that is distributed mainly in Ghana, Benin and western Nigeria (i.e. the type locality represents the westernmost record of this lineage).

(MOTU 13) *Mus* cf. *baoulei* “West”

Specimens from this lineage were found in Guinea and single individuals were sequenced from the eastern Ivory Coast [12] and Ghana [23]. Future more-detailed studies (using more samples, morphology and nuclear markers) are required to resolve whether MOTUs 12 and 13 represent separate species.

(5) The sorella group (Figure 1d):

It is a lineage of relatively large animals living in the Congo Basin’s forest-savannah transit zones, but also reported from south-eastern Africa (Mozambique and Zimbabwe) [57]. While very limited genetic data are available, our sampling shows very divergent sequences that may represent up to four species, but more data are required for taxonomic revision of this group.

(MOTU 14) *Mus* sp. “Dakawa”

Two sequences from Dakawa (Tanzania) belong to the *M. sorella* group, but they are very distinct from other lineages of the group (K2P distance = 8.74-9.75%). It is possible that they represent a new species, but more taxonomic research is necessary. There is an existing name, *M. wamae*, that may be valid for this MOTU. This taxon was described as a member of the sorella group from the Kapiti Plains in southern Kenya [57].

(MOTU 15) *Mus* sp. “Koi River”

A single specimen from the moist savannah area near Koi River in south-western Ethiopia clearly belongs to the sorella group, but is very divergent at *CYTB* (K2P-distance between MOTU 15 and other lineages of the sorella group are 9.72-9.83%). Further taxonomic work is necessary to resolve the taxonomic rank of this lineage. This is the first record of the sorella group in Ethiopia.

(MOTU 16) *Mus sorella* (Thomas, 1909)

The first sequence of this MOTU was published under the name *M. sorella* by [58] from Sangba (CAR). The species *M. sorella* was described from hills around Mt. Elgon, an area which has clear biogeographical connections to CAR (see e.g. MOTU 10 or clade C of MOTU27; Figure 1c and f). We obtained one additional short sequence from this lineage by 454 pyrosequencing of a museum specimen from the Garamba National Park in north-eastern DRC, thus connecting Sangba with the type locality. However, it is also possible that these sequences represent another currently valid species described from CAR, i.e. *M. oubanguii* Petter & Genest, 1970 or *M. goundae* Petter & Genest, 1970. More samples and detailed analyses are required to resolve this taxonomic problem.

(MOTU 17) *Mus neavei* (Thomas, 1910)

Even if more morphological comparisons are necessary, hereafter we call this south-east African clade *M. neavei* and we report the first sequences of this species. The type locality of *M. neavei* (also morphologically belonging to the sorella group; [57]) is Petauke, Zambia. In our material, this taxon is distributed in hilly areas of southern Tanzania, Malawi and one locality in Zambia (not far from the type locality). It occurs in sympatry with MOTU 6 from the triton group [57] and in the Nyika Mountains in Malawi also with MOTU 1. The records from South African Republic (SAR) are not yet confirmed genetically; the specimen mentioned by [14] was finally identified as *M. minutoides* and no other sequences of *M. neavei* were obtained despite intensive recent sampling efforts in SAR (F. Veyrunes, pers. comm.)

(6) The minutoides group (Figures 1e-f):

This is the most diversified group within *Nannomys*, inhabiting various, mostly open habitats of sub-Saharan Africa. It harbours the real “pygmy” mice, i.e. the rodents with the smallest body size (some of them with body mass < 5 g). Most previous published genetic studies of *Nannomys* mainly targeted representatives of this group. Our phylogenetic analysis reveals three clear subgroups: subgroup 1 (MOTUs 18 to 20), subgroup 2 (MOTUs 21 and 22), and subgroup 3 (MOTUs 23 to 27).

(MOTU 18) *Mus* sp. “Zakouma”

A single specimen of this taxon was captured in the Zakouma National Park in south-eastern Chad [11]. It is genetically very distinct from its sister species, *M. mattheyi* F. Petter, 1969 and *M. haussa* (Thomas & Hinton, 1920), and further taxonomic work on more material from southern Chad may confirm it as a new distinct species. Together with *M. mattheyi* and *M. haussa*, this species forms a monophyletic group that diverged in West African savannahs.

(MOTU 19) *Mus haussa* (Thomas & Hinton, 1920)

It is a Sahelian taxon, recorded in the belt from Senegal to western Chad [9]. Similarly as in *M. mattheyi* and other West African savannah species of rodents [59-61], there is also indication of longitudinal genetic structure in *M. haussa*, but more detailed data are needed for more conclusive phylogeographical inferences.

(MOTU 20) *Mus mattheyi* F. Petter, 1969

*M. mattheyi* is typical species of Guinean savannah-forest mosaic from westernmost Africa (Senegal) to the Dahomey gap, the relatively dry region separating Guinean and Congolese forest blocks [9]. It is divided into western and eastern phylogeographic subclades with a presumable contact zone in the Ivory Coast (not shown). It is often the most abundant *Nannomys* in the rodent assemblages [13,23].

(MOTU 21) *Mus* cf. *kasaicus*

Two sequenced individuals from the Kikwit region (DRC) formed this genetically very distinct genetic MOTU. There are also indications from other rodent groups that the Kikwit area is a local centre of endemism (see e.g. MOTU 5 or [62]). There is an existing name, *M. kasaicus* (Cabrera, 1924), described from Kasaï Occidental Province, Kananga, DRC, for the taxon belonging morphologically to *M. minutoides* group [10], that may apply to this MOTU.

(MOTU 22) *Mus indutus* (Thomas, 1910)

*M. indutus* is a south African species, found in a relatively large area from northern Botswana to southern SAR [11,14,63,64]. Records from Zambia and Malawi are based on genotyping of old museum material [64] and should be taken with caution. It is probably sympatric with *M. minutoides* Smith, 1834 (= MOTU 27) in most of its distribution range.

(MOTU 23) *Mus* cf. *gratus*

Specimens from this taxon were typically captured in forest clearings and the ecotone between forest and open habitats in equatorial Africa. There are three distinct clades with clear west–east geographical structure: (i) a single specimen from lowland tropical forest in Congo (K2P distance to two remaining clades is cca 7%); (ii) the Kisangani region in DRC; and (iii) both montane and lowland tropical forests in southern Kenya and northern Tanzania. More taxonomic work is necessary to link this clade to an existing species; possibly *M. gratus* (Thomas & Wroughton, 1910), a taxon from the minutoides group described from eastern Ruwenzori, “upper Congo” and Virunga mountains. Again, the comparison with the types will be required to verify this hypothesis.

(MOTU 24) *Mus* cf. *gerbillus*

This taxon is distributed in dry Somali-Maasai savannah in Kenya and Tanzania. In all phylogenetic analyses, it is a sister clade to the Ethiopian MOTU 25 (mean K2P distance between these two clades is 8.87%). Further taxonomic work is necessary, but *M. gerbillus* (G.M. Allen & Loveridge, 1933) (currently the synonym for Tanzanian populations of *M. tenellus*) is an available name that may apply to this lineage.

(MOTU 25) *Mus* cf. *tenellus*

This lineage was found at two close localities in northern Ethiopia - in Hagere Selam and in the Mekelle University campus. It may represent true *M. tenellus* (Thomas, 1903) described from Blue Nile in Sudan, but the comparison with the type material is necessary. On the contrary, morphological studies of museum material suggested that most published Ethiopian records of *M. tenellus* were actually *M. minutoides* [10].

(MOTU 26) *Mus musculoides* Temminck, 1853

It is a typical species of the Sudanian savannah belt. It was previously reported from western Africa [11,12,17] and northern Cameroon [65]. We provide a new very distant record from western Ethiopia, representing the easternmost genetically confirmed locality of the species. Very probably it is also present in poorly sampled countries such as Chad, northern CAR and South Sudan.

(MOTU 27) *Mus minutoides* Smith, 1834

**Figure 4:**
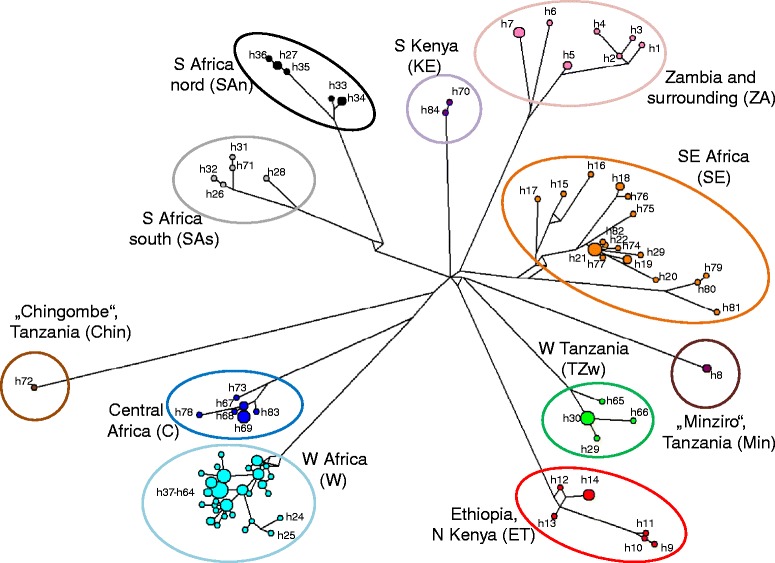
**Phylogeographical structure of** ***Mus minutoides*** **(MOTU 27).** Haplotype network was constructed by the median-joining algorithm from 131 *CYTB* sequences (84 haplotypes) in the program Network. The circle size is proportional to haplotype frequency and the connecting lines are proportional to number of substitutions.

*M. minutoides* is a widely distributed species in most of sub-Saharan Africa (probably except continuously forested areas in the Congo Basin and deserts; Figure 1f). This MOTU also includes specimens from southern Ethiopia; some of them were previously called *M. tenellus* [9,10]. This species has a very strong intraspecific phylogeographical structure. Median-joining network analysis of 131 sequences from this MOTU resulted in 84 haplotypes that form 11 strongly delimited haplogroups (Figure 4). The mean K2P-corrected distances among haplogroups ranged from 1.21% (TZw vs. KE) to 3.65% (ZA vs. Chin). All haplogroups are connected in the form of a star, suggesting multiple synchronous vicariance events. Allopatric divergences with subsequent expansions are further supported by current parapatric distribution of most clades and frequent, but narrow, secondary contacts among them (Figure 1f). The geographic structure within individual haplogroups is relatively weak, except clade SE, where it is possible to distinguish the separate sublineages from South Africa (h79-h81), Mozambique (h15-h17) and Tanzania (remaining haplotypes). Two haplogroups are only represented by animals from single localities (Minziro and Chingombe in Tanzania), but it is possible that they are more widespread in neighbouring regions in eastern DRC, where the relevant samples are missing.

### Divergence dating

The basal split of the extant *Nannomys* was dated at 5.24 Mya with 95% of the highest posterior density (HPD) between 4.58–5.96 Mya. Successive divergence of the extant major species groups then took place throughout Pliocene, with median estimates of divergence times ranging from 4.9 Mya (split off of MOTU 1 “Nyika”) to 2.44 Mya, i.e. the divergence of MOTU 23 (cf. *gratus*) and MOTUs 24–27 (i.e. four other species of the minutoides group) (Additional file 2). Posterior estimates of divergence dates at the calibration points are shifted towards past in the case of *Apodemus* (prior median 5.89, posterior median 7.38) and *Arvicanthis*-*Otomys* (6.81 vs. 8.13) but towards the present in the case of *Mus* (8.00 vs. 7.44). Two other divergence dates are also worth noting: the split-off of *Myomyscus yemeni* estimated at 6.21 (5.12–7.33) Mya, which is consistent with its migration to Arabian peninsula across the land bridge during the Messinian crisis, and the origin of modern *Otomys* 3.77 (2.83–4.81) Mya, first appearing in the fossil record around 3 Mya [[66], p.290]. Complete results of the divergence dating analysis are reported in Additional file 2.

The full set of branching times between 27 MOTUs is given in Figure 3a. It is based on the secondary dating of the ultrametric tree for GMYC, but the posterior estimates of divergence dates are consistent with previous analysis (compare Figure 3a and Additional file 2). Main species lineages diverged in lower Pliocene (5.2-4.5 Mya) and an intensive period of speciation is also visible in the lower Pleistocene (2.1-1.6 Mya), when many extant lineages within main species groups appeared.

### Biogeographical analysis

Bayesian analysis of discrete traits in BEAST revealed that the most ancestral distribution (98% support) of *Nannomys* included mountains of Eastern Africa (Figure 3b). This type of distribution is currently present in all three ancient monotypic lineages (MOTUs 1–3), as well as in numerous lineages of the triton and setulosus groups. There are two major habitat shifts in the *Nannomys* evolution. (1) The lineage leading to the baoulei group colonized the forests (and forest-savannah mosaic) in western Africa cca 4 Mya, where it split to western and eastern sublineages later on; (2) the minutoides group descended from mountains, adapted to more arid open habitats, and started to radiate across the whole sub-Saharan Africa cca 3.5 Mya. In the first radiation phase, MOTUs 18–27 speciated in savannah-like habitats over all of Africa (approx. 3.5-1.6 Mya). Geographically similar, but much more recent (cca 1 Mya) radiation occurred inside MOTU 27, i.e. *M. minutoides* (Figures 1f, 3a, and 4).

Very similar results were obtained by the maximum likelihood approach in Lagrange (Additional file 5). Most basal splits occurred with the highest probability in the mountains of East Africa, also where most of the MOTUs from the triton group diverged. The first clear shifts to other habitats are visible in the ancestors of the baoulei group (to the forests or forest edges, where both MOTUs from this group occur until today) and in the ancestors of the minutoides group (to the savannah). The most intensive radiation in the latter took place in savannahs, with one shift to the forest habitat detected in MOTU 23 (*M.* cf. *gratus*). The estimates of ancestral ranges are less clear in the setulosus and the sorella groups. While the former started to diverge most probably in mountains (with subsequent spreading of two “*setulosus*” MOTUs to forests of central and eastern Africa), the latter had ancestors occurring with similar support either in savannahs or in hills of Eastern Africa.

## Discussion

For the purpose of our study, we compiled new and existing sequences into the largest genetic dataset to date of the subgenus *Nannomys* and performed the first phylogenetic analysis of the group that contains most of the currently recognized valid species across the whole sub-Saharan Africa. We detected a surprisingly high amount of cryptic diversity, with numerous candidates for new species. Wide geographical sampling also allowed the first empirical definition of the distribution areas of all the detected lineages based on physically present genotyped individuals. Using several calibration points and the current distributional data, we also carried out biogeographical analysis and reconstructed the possible evolutionary scenario of this highly successful group of sub-Saharan murines.

### Species concepts and estimation of the number of *Nannomys* species

Species diversity crucially depends on the adopted species concept. Widely used concepts of typological or biological species are not always applicable for species delimitation because of frequent convergent evolution, cryptic species, and the impossibility of proving reproductive isolation among allopatric populations. Together with the rapidly increasing amount of genetic data from free-living populations, these concepts are often complemented by genetic [37] or phylogenetic [3] species concepts, creating the so-called integrative taxonomic approach. Although genetic approaches can sometimes lead to an unjustified increase in the number of species (so-called taxonomic inflation [2,67]), they often detect cryptic diversity within evolutionary lineages that can be generally important from the taxonomic as well as conservation point of view. In our study, we used the combination of maximum likelihood delimitation of phylogenetic species and the genetic distances to estimate the number of MOTUs (= putative species) of *Nannomys* in Africa. We are aware of the drawbacks of these approaches (e.g. the use of only maternally inherited mtDNA), however, our aim was not to perform the taxonomic changes based solely on limited genetic data, but rather to identify the taxa and regions of high cryptic diversity requiring more detailed taxonomic studies.

The combination of different approaches revealed the existence of 27 MOTUs. This is considerably more than the 18 currently accepted *Nannomys* species [9,10], suggesting that numerous putative species have so far remained undetected, and therefore undescribed. Most of the genetic data of *Nannomys* that have been collected to date originate from Western and Southern Africa, where the taxonomy of this group has been intensively explored (reviewed by [9]). The number of candidates for potential new species in western Africa revealed by our study is therefore relatively low (only *M*. cf. *setulosus* “West” or *M*. cf. *baoulei* “West”) and it is also possible that these MOTUs just represent marked phylogeographical structure of within-species lineages with parapatric distribution (but see [12] that already suggested *M. setulosus* as a species complex).

The situation is completely different in Eastern Africa, from where only fragmentary genetic data were available prior to this study. Our results may lead to the description of more than 10 new species that are already now sufficiently delimited using the combination of genetic, ecological and geographic data. Many of these so far undescribed taxa occur in mountains or highland habitats, but a few other potential new species (like *M.* cf. *gerbillus*) are typical inhabitants of dry savannahs. The taxonomic diversity of *Nannomys* is probably the highest in Ethiopia. As for many other organisms, the Ethiopian highlands represent an important hot-spot of African endemism for *Mus*. We have revealed the presence of 8 MOTUs in this country, and only two of them (*M. minutoides* and *M. musculoides*) have also been recorded outside Ethiopia. The six remaining species are probably endemic, making *Mus* the genus with the second highest number of Ethiopian mammal endemics (after *Lophuromys* with 9 endemic species; [68]).

Even if we have not sequenced the type material of most currently valid taxa (except paratypes of *M. bufo*), we have been able to assign the most probable species names to 13–14 MOTUs based on previous genetic studies (including karyotypes; [11,12]), geographical distribution (i.e. sequences from the type locality or close neighbourhood) and external morphology. Therefore, the genetic dataset from this study represents a solid basis for future identification of morphologically similar *Nannomys* species via DNA barcoding (using e.g. evolutionary placement algorithm; [69]). Unfortunately, our dataset lacks sequences of four valid species. *M. oubanguii* Peter & Genest, 1970 and *M. goundae* Peter & Genest, 1970 represent two species from the sorella group known only from few localities in the Central African Republic. They were described mainly on the basis of external morphology [57] and their specific status has been questioned previously ([10]; but see conspicuous differences in karyotypes of these two species - reviewed in [9]). The whole sorella group requires a profound revision including new sampling in savannahs north of the Congo Basin and additional genetic data. We found high genetic variation within the sorella group, but most clades are represented by only one or two localities (except *M. neavei*) and in most cases it is not possible to assign the particular clades to currently valid species names. *M. setzeri* Petter, 1978 is a rare taxon with limited distribution in dry areas of Namibia, Botswana and western Zambia [70,71]; it is probably a valid species as it can be morphologically distinguished from sympatric *Nannomys* species [63,72]. *M. orangiae* Roberts, 1926 is the fourth species that is currently valid and missing from our dataset. It also is a southern African species with unclear taxonomical status. It was previously considered a subspecies of either *M. setzeri* or *M. minutoides* [10] and may just represent one of the cytotypes of the latter [9].

Phylogenetic estimate of species richness of *Nannomys* in our study (25–30 MOTUs that may represent separate species) suggests that it is one of the most speciose groups of African terrestrial mammals. Similarly well studied species-rich genera of African rodents usually have a lower number of monophyletic genetic lineages considered as species, e.g. *Praomys* (16–20 species; [73]) or *Hylomyscus* (21 species, including undescribed and recently described taxa; [74], J. Kennis et al., submitted). The only genus with higher described species richness is *Lophuromys* (29 species; [10,68,75]). However, this genus is specialized to tropical forests and ecotones and it is likely that intensive genetic drift in fragmented forest habitats (especially in Eastern Africa) caused morphological distinctiveness allowing differentiation of a high number of genetically similar morphospecies [68,75]. It is also worth to note that in comparison with the above-mentioned genera, *Nannomys* colonized a much wider spectrum of habitats (from Afroalpine meadows and mountain forests to very arid savannah).

### *Mus minutoides* as a model for pan-African phylogeography

The MOTU with the largest distribution of all *Nannomys* is *M. minutoides* (=MOTU 27). There are only a very few such widespread savannah-forest mosaic species distributed across almost complete sub-Saharan Africa. Among rodents, only the ubiquitous *Mastomys natalensis* had held this habitat breadth, and it was considered the rodent species with the largest distribution area in Africa [10]. Our genetic data confirm that *M. minutoides* has very similar and probably even larger distribution than *M. natalensis*. It can be argued that MOTU 27 does not represent a single species but rather a species complex, which may be supported by the GMYC analysis revealing significant support for additional speciation events within this clade (see Figure 3a). However, in absence of more detailed evidence, we prefer to maintain all genetic lineages of MOTU 27 within the species *M. minutoides*. They do not show visible external differences (although detailed morphological analysis of genotyped material is still missing), they radiated relatively recently (last 1 Mya) and the Tamura-Nei corrected genetic distances among clades (1.21-3.65% on *CYTB*) are comparable with those among clades of *M. natalensis* (2.1-3.8%; [76]), i.e. much lower than usual genetic distances between sister species of rodents [37]. Further detailed studies should focus on the contact zones of divergent clades to reveal whether they can interbreed or not.

Species with large distributions and strong affinities to open habitats can serve as possible models for comparative pan-African phylogeography of the savannah-like biomes. Recent phylogeographic studies of *M. natalensis* showed that populations were strongly influenced by Pleistocene climate fluctuations [76]. The presence of genetically divergent clades with parapatric distribution is congruent with the scenario invoking allopatric fragmentation and vicariance. Almost the exact same geographic pattern of genetic differentiation is visible in *M. minutoides* (compare Figure 1f in this study with Figure 1 in [76]). The phylogeographic pattern suggests at least 11 different savannah refugia approximately 1 Mya, i.e. in the period of very strong climatic instability [77]). The genetic lineages evolved in allopatry and subsequently spread during suitable periods of savannah expansion. Further research should focus on precise localization of refugia by combining information from population genetics with modelling of past ecological conditions [78]).

### A new biogeographical scenario of *Nannomys* radiation in Africa - from mountains to lowland forests, savannahs and arid Sahelian environments

More complete taxon sampling from the whole sub-Saharan Africa now allows significant modification and increased precision of the previously proposed biogeographical scenario of *Nannomys* radiation in Africa [11]. Our molecular dating based on plausible paleontological calibration and taxon-unbiased phylogenetic tree suggests that the divergence of the genus *Mus* to the current subgenera occurred in Asia in the late Miocene (cca 6.8-7.4 Mya), which is in good agreement with previous studies [7,11,44]. The colonization of Africa by *Mus* occurred very probably in the Messinian period (7.3-5.3 Mya) when the temporary land bridge connected Africa and southwest Arabia. In this period, many faunal exchanges between Africa and Asia are well documented [79-81]. It is therefore highly probable that *Mus* was already in Africa at the beginning of the Pliocene. The basic split of the extant *Nannomys* was dated at 5.24 Mya (95% HPD 4.58–5.96 Mya), i.e. very soon after a land bridge between Africa and Southwest Arabia disappeared (5.3-6 Mya; [82,83]). The oldest fossil evidence of the genus *Mus* in Africa was from the early to middle Pliocene in Ethiopia (the Omo valley in the south of the Ethiopian Rift Valley and Hadar in the east, 5–2.5 Mya; [84,85]) and Kenya (4.5 Mya; [27]).

Due to incomplete sampling (mainly in eastern Africa) previous studies could not adequately explore the evolutionary history of *Nannomys*, especially since our biogeographical reconstructions demonstrate that the first divergence of *Nannomys* occurred in eastern Africa. Paleoclimatic and paleoanthropological research in eastern Africa suggested repeated association of critical events in hominin evolution with the most prolonged intervals of high climate variability. Potts (2013) [77] defined eight intervals of predicted high climate variability in the last 5 My and argued that most important events in hominin evolution occurred within these periods. Three of the most prolonged intervals of predicted high climate variability are 2.79-2.47 Ma, 1.89-1.69 Ma, and 1.12-0.92 Ma and they largely overlap with the previously defined periods of the occurrence of large lakes [86] as well as with inferred aridity phases based on dust records, paleosol δ13C, and the prevalence of grazing bovids [87].

Clear associations between periods of climatic instability and divergence events are also visible in phylogenetic reconstructions of *Nannomys*. The first splits leading to ancestors of most current species groups probably occurred in eastern Africa in the period 5.2-4.5 Mya (Figure 3a), which corresponds to the longest era of strong wet-dry variability [77]. Nothing is known about the ecology of the extinct *Mus* taxa, but surviving ancient lineages (MOTUs 1–3) may provide some clues. They can be considered “living fossils”, i.e. monotypic relict taxa living in very restricted areas in Eastern African mountains. The period 4–3.5 Mya is considered relatively stable with few documented evolutionary events [77] and we observed only two vicariance events in *Nannomys* during this period. The first is the north–south split of MOTU 3 (*M*. sp. “Harenna”) and the triton group, and the second is the west–east split of the baoulei and the sorella groups (see Figure 3a and compare it with distributions at Figure 1). The most intensive radiation of *Nannomys* is dated into the period 3.5-1.4 Mya (see Figure 3a), when most current MOTUs (i.e. putative species) appeared. The beginning of this period coincides with the start of a cooling and aridification trend [88]. The open savannah-like habitats were spreading intensively and at the same time the climate was very variable (four prolonged periods of strong wet-dry variability are dated into this range; [77]). This variable climate likely yielded environmental changes that increased the frequency of evolutionary responses like adaptation, dispersal (especially in open habitats), and ultimately, speciation (for example it was also the period with the highest number of hominin taxa; [89]). Our biogeographic analyses are consistent with these findings because the most intensive radiation occurred in the minutoides lineage in savannahs. The presumed shift from mountains to more arid and open habitats was clearly linked with the decrease of the body size in the minutoides lineage. The ancient *M. imberbis* (MOTU 2) has a body size of 25 g [8], MOTU 1 (*M*. sp. “Nyika”) has 14 g, MOTU 3 (*M*. sp. “Harenna”) has cca 16 g (our unpublished data) and the members of other non-*minutoides* groups weight 8–13 g [9]. In contrast, all species of the *minutoides* clade have body size 3–8 g, making them one of the smallest mammals in the world [9]. The last period of climatic instability is dated to 1.12-0.92 Mya, which coincides with the likely simultaneous split of the MOTU 27, i.e. *Mus minutoides*, into 11 distinct genetic lineages (see above).

### Ecological constraints and multi-species sympatry

Previous studies revealed that at several sites more than one species of *Nannomys* occurs in sympatry [12,13,23]. Their observations are in agreement with the distribution ranges based on genotyped individuals (Figure 1) showing largely overlapping distribution areas of many species. However, if we exclude widely distributed *M. minutoides* (MOTU 27), the distribution of individual species within the same species group is predominantly parapatric (most illustrative in Figure 1b, c, d), while sympatry is typical for species from different species groups. This suggests that the species groups might have evolved specific morphological adaptations that allow their sympatric occurrence with the members of other *Nannomys* lineages. Although detailed morphological analysis of genetically identified specimens is still missing, preliminary data suggest clear differences in the skull morphology among the species groups ([12], E. Verheyen et al., unpublished data), with possible functional consequences in separation of ecological niches (for example dietary).

Even if the distribution areas of two or more species from the same species group overlap, closer examination of our data provide evidence for the preference of different habitats. For example two Ethiopian endemics from the setulosus group, *M.* cf. *proconodon* (MOTU 7) and *M. mahomet* (MOTU 11), have never been captured at the same locality; the former prefers lowland habitats in the Rift Valley, while the latter is common species across the Ethiopian highlands. Up to four species of the minutoides group can be found in western Africa, but their ecological requirements are probably different. Based on the data summarized at Figure 1 and published records, it seems that *M. minutoides* is able to live in Western Africa in relatively humid places, *M. mattheyi* prefers dry Guinean savannah and the transition zone between forest and savannah, *M. musculoides* is a typical inhabitant of Sudanian savannah belt from Guinea to western Ethiopia and *M. haussa* lives in arid Sahelian environment ([12,65], figure one in this study). Similarly *M. minutoides* can occasionally be found in the same localities as *M. indutus* in southern Africa, but the latter probably prefers drier habitats ([63,64] and references therein).

### Relevance to the understanding of karyotype evolution and sex determination

The subgenus *Nannomys* has previously been used as a suitable model for studies of karyotype evolution due to very high variability of chromosomal rearrangements [11,17,90,91]. The ancestral karyotype of the pygmy mice was composed of 36 acrocentric chromosomes [17,92], but the wide spectrum of mutational mechanisms modified the chromosomal constitution. Besides relatively frequent Robertsonian translocations, other chromosomal rearrangements were described in *Nannomys*, including variable sex-autosome translocations, pericentric inversions, tandem fusions and WARTs (Whole-Arm-Reciprocal Translocation) [9]. Pan-African phylogeny based on more complete taxon sampling presented in our study can help to understand the karyotype evolution in general and sex determination mechanisms in particular. The mapping of karyotype features on the phylogenetic tree can help to define specific predictions that can be further verified by sampling focussed on particular species and geographical areas.

For example, tandem fusions - one of the rarest chromosomal rearrangements - were evidenced in *M. triton* (MOTU 4) and *M.* sp. “Harenna” (MOTU 3) that in most phylogenies cluster together. Even if they were suspected in two other species of the sorella group in the CAR (*M. goundae* and *M. oubangui*, not sampled in our study; [9]), further detailed studies of these rare mutations should direct their focus on widely distributed and common MOTU 6 belonging to the triton group. One of the most conspicuous features of *Nannomys* karyotypes is the fusions of autosomes and sex-chromosomes. These fusions were most frequently studied in two terminal taxa of the minutoides group (MOTU 26 - *musculoides* and MOTU 27 - *minutoides*), but they were also observed in *M. goundae* and *M. oubangui* (very probably belonging to the sorella group) and in *M. triton* (MOTU 4) [9]. Since they appeared several times independently, it is therefore clear that predispositions for translocations of sex chromosomes exist in more lineages of *Nannomys*, yet these translocations are not a general feature of the whole subgenus, as, for example the setulosus group is very conservative and all MOTUs karyotyped until today have the ancestral karyotype (2n = 36, NF =36) ([78] and references in [9]). Future research on East African species of the minutoides group (MOTUs 23–25, i.e*. M.* cf. *tenellus*, *M*. cf. *gerbillus*, and *M.* cf. *gratus*), the sorella group and the triton group could thus potentially bring interesting new insights on the evolution and polymorphism of sex-autosome translocations. Finally, the phylogeographic pattern described in our study for the most karyotypically variable species, *M. minutoides*, can help to design further sampling of chromosomal data in lineages, where the karyotypes are not yet known. The haplotype network suggests 11 main lineages that probably differentiated in small allopatric populations at the same time, which could have led to establishment and fixation of important karyotypic differences [64,90]), possibly involving presently unknown means of sex determination [91]. If such karyotype differences among genetic lineages exist, it would be also extremely interesting to study the possible contact zones among them (see Figure 1f).

### Consequences for future epidemiological studies

Rodents are reservoir hosts of important human pathogens, of which some can cause serious diseases. Most recent examples of emerging and re-emerging diseases have been caused by RNA viruses [93] and understanding of their evolution and epidemiology is essential for predicting future emergences and designing interventions (e.g. vaccinations). Among RNA viruses hosted primarily by African rodents, the Lassa arenavirus has received most attention, because it is responsible for Lassa hemorrhagic fever in West Africa, which causes thousands of human deaths each year [94]. The host specificity of arenaviruses is thought to be relatively strict with often a single species described as the primary reservoir host [21]. A long-term evolutionary history between arenaviruses and their hosts (co-evolution) was originally suggested due to the almost perfect sorting of arenavirus lineages into rodent clades (e.g. [95]). Recent studies suggest that pygmy mice are frequent hosts of arenaviruses (and probably also other important parasites) and they often live close to human habitations (e.g. [13]). The pan-African phylogeny of *Nannomys* proposed in this paper can help to describe the co-evolutionary patterns of arenaviruses and their hosts and even provide the potential clues for understanding the occasional disease outbreaks.

The first virus found in *Nannomys* was the virus Kodoko, described from *Mus minutoides* in western Africa, and belonging to the lineage of lymphocytic choriomeningitis virus (LCMV) that is hosted by the house mouse [20]. This finding (followed by description of new Kodoko strain in Eastern African, [21]) thus supported a co-evolutionary scenario, because all arenaviruses known from African murine hosts at that time grouped according to taxonomic position of theirs hosts (i.e. three main lineages of African arenaviruses were hosted by rodents of three tribes, Praomyini, Arvicanthini and Murini; Lecompte et al. 2007, Gouy de Belocq et al. 2010). However, the next arenavirus, called Gbagroube and described from *Mus* cf. *setulosus* (MOTU 9 in this study) from the Ivory Coast, does not belong to the LCMV lineage (specific to *Mus*), but surprisingly clusters with the Lassa virus strains [22]. Very recently, two other arenaviruses were found in *Nannomys* in Ghana [23]. The virus Natorduori is hosted by *M. mattheyi* (MOTU 20, the minutoides group) and clusters clearly into *Mus*-specific LCMV lineage. In contrast, the virus Jirandogo, the first arenavirus reported from *M. baoulei*, in various phylogenies based on its different genome segments belongs to the Lassa virus group (similarly as Gbagroube virus). African pygmy mice are therefore the first group of African rodents that host two very different lineages of arenaviruses; one of them seems to be *Mus*-specific (in Africa now reported from two species in the minutoides group), but the second forms the sister lineage of the highly pathogenic Lassa virus (hosted by species from the setulosus and the baoulei groups). Further surveillance for new arenaviruses focussed preferentially on *Nannomys* lineages where no viruses have yet been found (e.g. the triton or sorella groups widely distributed in central and eastern Africa) can increase understanding of the evolution of these pathogens and predict the regions of possible epidemiological importance.

## Conclusions

The known species diversity of tropical organisms is highly underestimated even for relatively well known animals like mammals. Here we performed a phylogenetic analysis of the largest available set of genetic data collected from the only indigenous African lineage of the genus *Mus*, called *Nannomys*. A conservative definition of MOTUs suggests that the number of species described to date represents only approximately 60% of possible species diversity and intensive taxonomic work is now required to allow the formal description of genetically divergent lineages. We also provide the first reliable genotype-based distribution ranges of particular MOTUs that can aid in future species inventories in different parts of Africa. The dating of divergences and biogeographical analyses strongly suggest that ancestors of *Nannomys* colonized Africa at the end of Miocene and diverged to ancestors of the main species groups in mountains of Eastern Africa in lower Pliocene. The aridification that started in Africa cca 3 Mya led to spreading of open habitats and provided new ecological niches that were fully utilized by *Nannomys*. In particular, the so-called minutoides lineage underwent an exceptionally intensive radiation in savannah-like habitats and occupied almost whole sub-Saharan Africa in several colonization waves. The combination of a detailed phylogeny based on an almost complete taxon sampling combined with genotype-based distributional data of lineages, taxa and valid species provides a solid foundation to address specific ecologically-explicit evolutionary hypotheses using *Nannomys* as a model system, i.e. in evolution of sex determination and host-virus co-evolution.

### Availability of supporting data

The newly produced sequences were submitted to GenBank under accession numbers KJ935741-KJ935873 (*CYTB*) and KJ935874-KJ935905 (*IRBP*) (see Additional file 1 for more details). The final alignment of concatenated sequences used in phylogenetic analyses is in Additional file 6.

## Additional files

Additional file 1:
**Details on collecting localities and genetic data for all** ***Nannomys*** **specimens.**
Additional file 2:
**List of sequences used for divergence dating and resulting fossil-calibrated timetree.**
Additional file 3:
**Maximum likelihood phylogeny of** ***Nannomys*** **based on separate analyses of mitochondrial** ***CYTB*** **and nuclear** ***IRBP*** **genes.**
Additional file 4:
**Distribution of genetic distances at** ***CYTB*** **within and among taxa delimited by different methods.**
Additional file 5:
**Biogeographical reconstruction of ancestral distribution of** ***Nannomys*** **lineages using maximum likelihood in Lagrange.**
Additional file 6:
**Alignment of 179 ingroup and 7 outgroup concatenated sequences of** ***CYTB*** **and** ***IRBP.***

## Abbreviations

*CYTB*: Mitochondrial gene for cytochrome *b*
IRBP: Gene for interphotoreceptor binding protein
Mya: Million years ago
mtDNA: Mitochondrial DNA
ML: Maximum likelihood
BI: Bayesian inference
MOTU: Molecular operational taxonomic unit
GMYC: Generalized mixed Yule-coalescent model
PCR: Polymerase chain reaction
AIC: Akaike information criterion
LCMV: Lymphocytic choriomeningitis virus
