# Supplementary material for: Pan-African phylogeny of Mus (subgenus Nannomys) reveals one of the most successful mammal radiations in Africa
Source: BMC Evol Biol. 2014 Dec 14;14:256. doi: 10.1186/s12862-014-0256-2 (PMC4280006; doi:10.1186/s12862-014-0256-2)

Additional File 2: List of sequences used for divergence dating and resulting fossil-calibrated time-tree.

Supplementary Table: Data set used for the divergence dating analysis in BEAST. GenBank accession codes for *IRBP* and *CYTB* sequences are shown, as well as ID of the voucher specimens (when available).

Taxon	IRBP	CYTB	Voucher (IRBP/CYTB)
Aethomys	AY326075	AJ604526	FMNH168101/TZ337
Apodemus agrarius	AB096842	AB096809	HS1733
Apodemus argenteus	AB032855	AB032848	HS361
Apodemus draco	AB109398	AB109397	KIZ211058
Apodemus flavicollis	AB032860	AB032853	HS591
Apodemus gurkha	AB032859	AB032852	HS1317
Apodemus latronum	AB096851	AB096834	HS2515
Apodemus mystacinus	AB303229	AJ748237	MAS2/JRM-544
Apodemus speciosus	AB032856	AB032849	HS240
Apodemus witherbyi	AB303231	AB303228	Her103
Arvicanthis neumanni	KC953358	EU349737	H 894
Arvicanthis niloticus	DQ022386	AF004569	MNHN 1997-1842
Colomys	DQ022395	AF518372	SMNS 30124
Dasymys	EU292143	AF141217	?
Golunda	AM408332	AM408338	T-843
Grammomys	AM408329	AM408345	T-1092
Heimyscus	DQ022397	AF518332	MNHN 2001-64
Hylomyscus parvus	DQ022399	AF518329	CM SP_10502
Hylomyscus stella	DQ022398	AF518331	CM SP_5032
Lemniscomys rosalia	DQ022390	AF141209	MNHN 1996-569/?
Lemniscomys striatus	AM408321	AF141210	T-789/?
Malacomys edwardsi	DQ022392	DQ022379	R24689
Malacomys longipes	DQ022393	DQ022380	MNHN_2001-070
Mastomys coucha	DQ022400	AF518334	MNHN 1999-104
Mastomys erythroleucus	AM408335	AM409395	T-295/M4912
Mastomys pernanus	DQ022403	AF518343	RUCA 1624
Micaelamys	AM408330	AF141215	T-1425/?
Mus cookii	AB125802	AB125767	HS2921
Mus musculus	AF126968	V00711	
Mus pahari	AJ698893	AY057814	
Mus crociduroides	AJ698894	AJ698878	T-1194
Mus platythrix	AJ698895	AJ698880	T-406
Myomyscus brockmani	DQ022407	AF518352	CM TK_33059
Myomyscus yemeni	DQ022409	AF518356	MNHN 1994-079
Oenomys	KC953425	EU349769	CM_102549/CM_102548
Otomys angoniensis	AM408325	AM408343	T-718
Otomys denti	KC953428	EU874449	FMNH_138124
Parotomys	KC953432	AF141224	H-656/?
Praomys daltoni	DQ022406	AF518348	MNHN 1999-392
Praomys degraafi	DQ022410	AF518359	FMNH 138046/FMNH 157790
Praomys delectorum	AY326104	KC261544	FMNH153977/RMCA 96.037-M-4755
Praomys jacksoni	KC953443	EU349778	CM 102583
Praomys petteri	JF284269	JQ735842	MNHN R13072
Praomys tullbergi	DQ022413	AF518365	CM SP10188
Praomys verschureni	DQ022394	AF518373	SMNS 131

Stenocephalemys albipes	DQ022404	AF518346	MNHN 1999-561
Stenocephalemys albocaudata	DQ022414	AF518369	T-1590
Stochomys	EU292147	EU292149	MNHN 1999-459
Tokudaia	AB033712	AB033703	HS1162
Zelotomys	DQ022396	AF518375	A. Hoffman 3191
MOTU 1 ("Nyika")	KJ935874	KJ935741	M8_3025_Nyika_MW
MOTU 2 (*imberbis*)	KF928334	KF928333	ETH121_Chilalo_ETH_imberbis
MOTU 3 ("Harenna")	KJ935875	KJ935743	ETH211_HarenaForest_ETH
MOTU 4 (*triton*)	KJ935748	KJ935876	KE913_Nanyuki_KE
MOTU 7 (cf. *proconodon*)	KJ935883	KJ935767	ETH299_MagoNP_ETH
MOTU 8 (*setulosus*)	AJ698888	AJ698873	AJ698873_GAB_setulosus
MOTU 12 (*baoulei*)	-	EU603991	BE55_Agbassa_BE
MOTU 17 (*neavei*)	KJ935890	KJ935805	M8_3049_Nyika_MW
MOTU 18 ("Zakouma")	AJ875089	AJ875085	AJ875085_Zakouma_TCH
MOTU 19 (*haussa*)	AJ875073	AJ698891	AJ875073_Kollo_NIG_haussa
MOTU 22 (*indutus*)	AJ698892	AJ698874	AJ698874_Kgalagadi_SA_indutus
MOTU 23 (cf. *gratus*)	KJ935894	KJ935822	TA77_Minziro_TZ
MOTU 27 (*minutoides*)	KJ935903	KJ935867	TA171_Rumanyika_TZ

Supplementary Figure: Dating of divergences of murine rodents using relaxed clock model with substitution rates drawn from an uncorrelated lognormal distribution in BEAST 1.8.0 (Drummond et al., 2012) and three fossil-based calibration points (marked by stars).

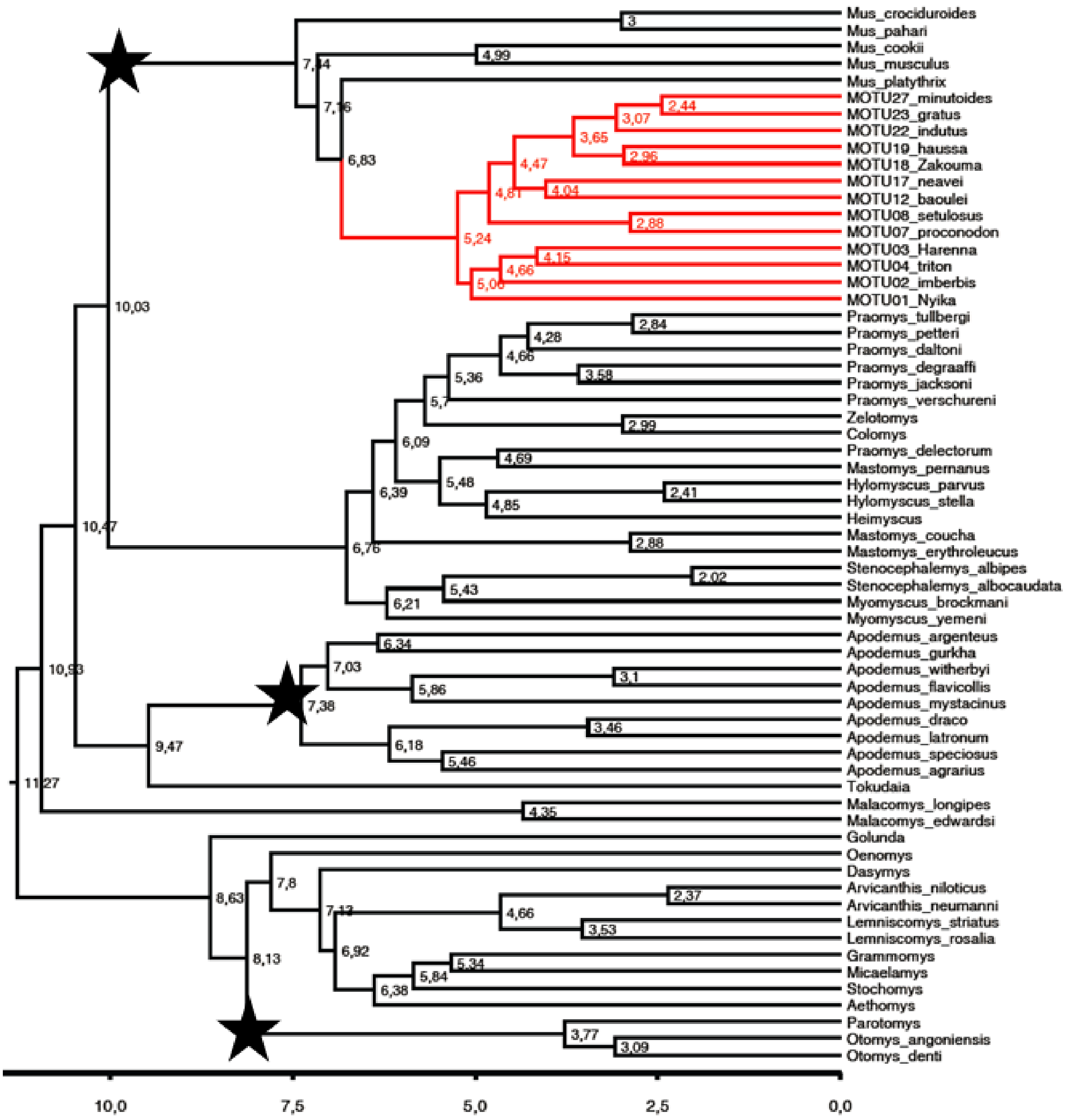

Supplement: Additional file 2: — List of sequences used for divergence dating and resulting fossil-calibrated timetree. [file 12862_2014_256_MOESM2_ESM.pdf]
