# Supplementary figures and images for: Pan-African phylogeny of Mus (subgenus Nannomys) reveals one of the most successful mammal radiations in Africa
Source: BMC Evol Biol. 2014 Dec 14;14:256. doi: 10.1186/s12862-014-0256-2 (PMC4280006; doi:10.1186/s12862-014-0256-2)

(A) ML tree of *CYTB*

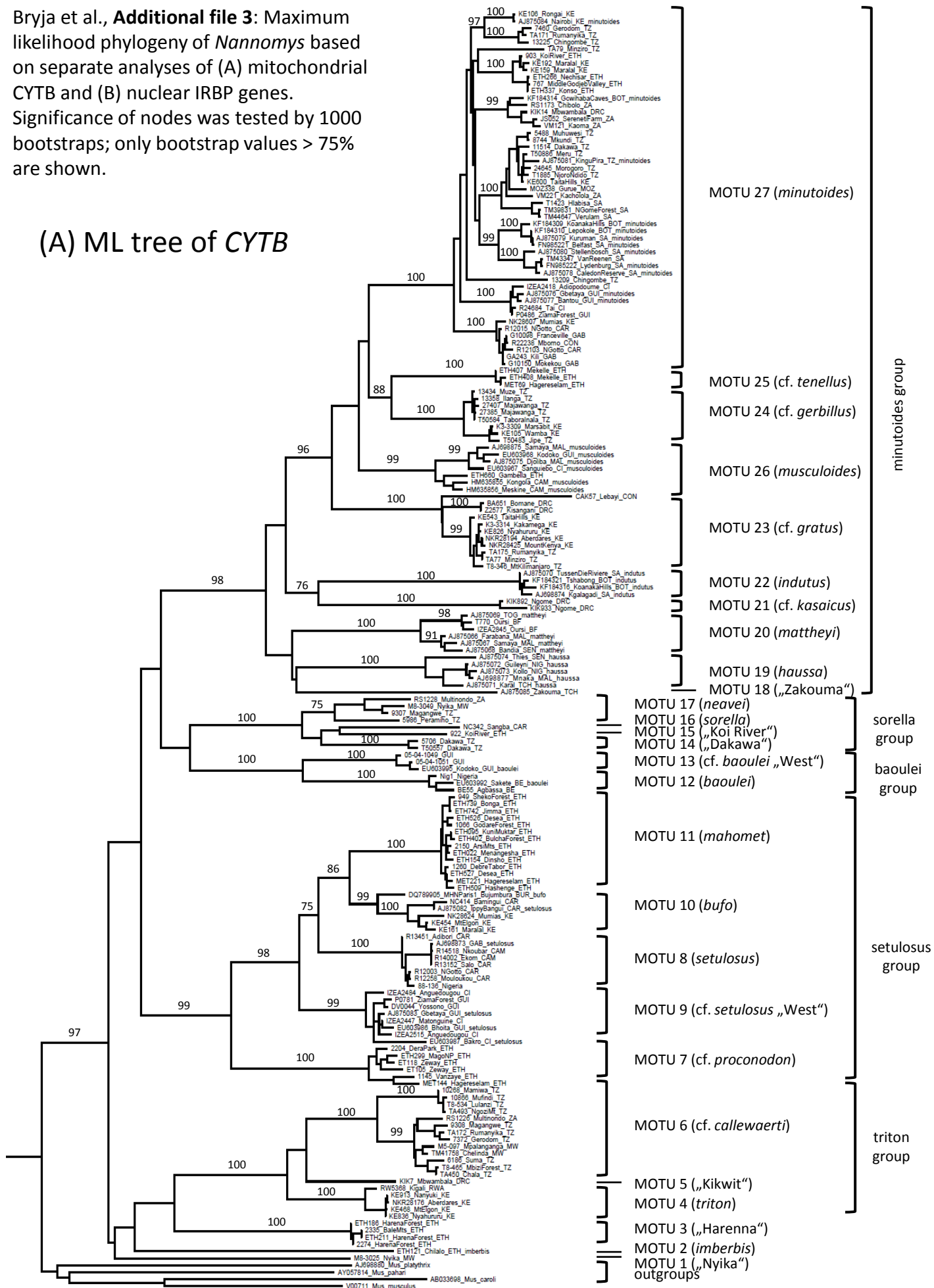

(B) ML tree of *IRBP*

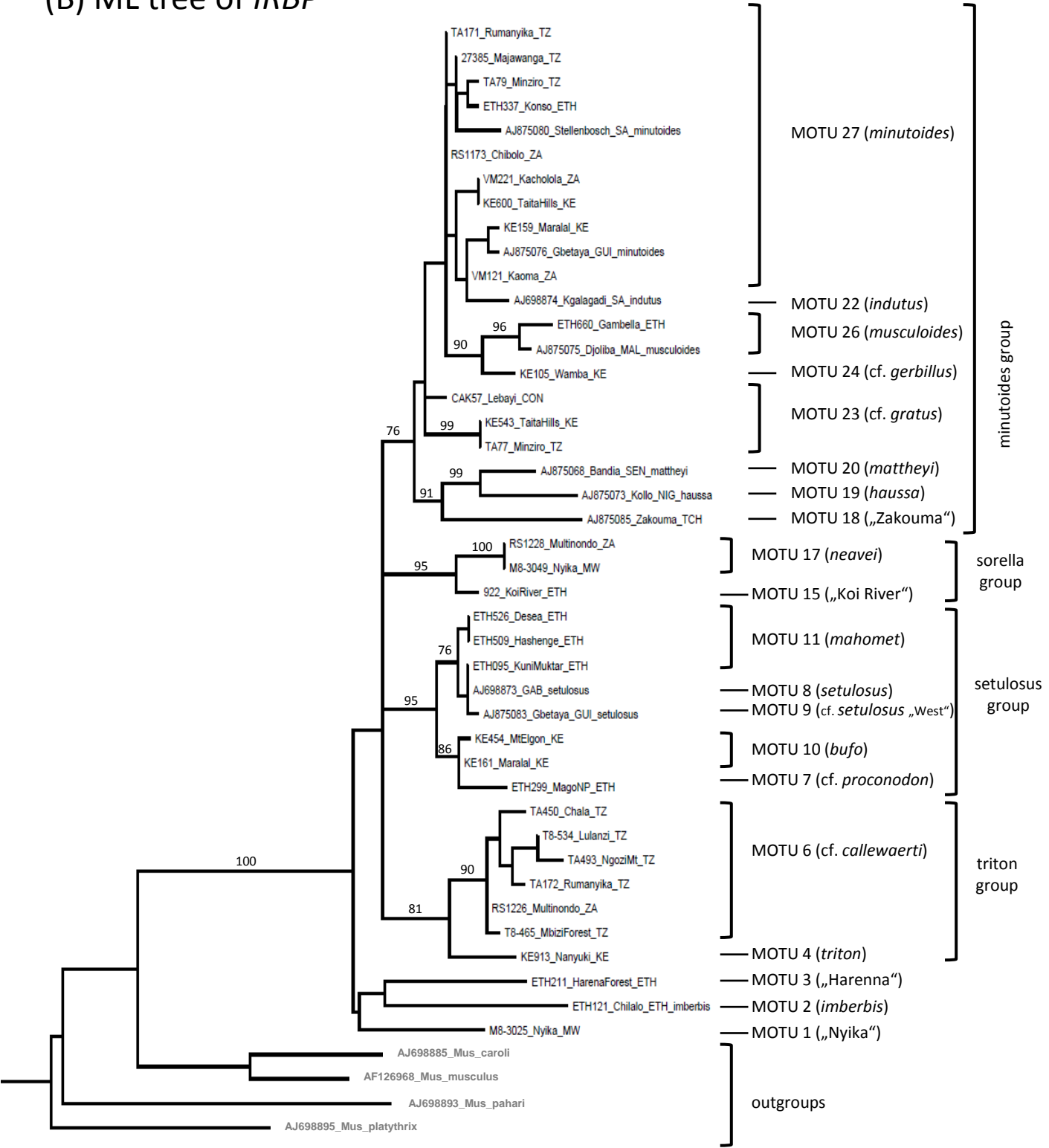

Supplement: Additional file 3: — Maximum likelihood phylogeny of Nannomys based on separate analyses of mitochondrial CYTB and nuclear IRBP genes. [file 12862_2014_256_MOESM3_ESM.pdf]
