# Supplementary material for: Pan-African phylogeny of Mus (subgenus Nannomys) reveals one of the most successful mammal radiations in Africa
Source: BMC Evol Biol. 2014 Dec 14;14:256. doi: 10.1186/s12862-014-0256-2 (PMC4280006; doi:10.1186/s12862-014-0256-2)

Bryja et al., **Additional file 4**: Distribution of K2-P distances within and among (a) GMYC-defined „species“ and (b) 27 MOTUs defined by the combination of genetic distances and geographic distribution (see the text for more details). Box-and-Whisker plots represent the median, range and quartiles of the mean K2-P distances within and between particular taxa.

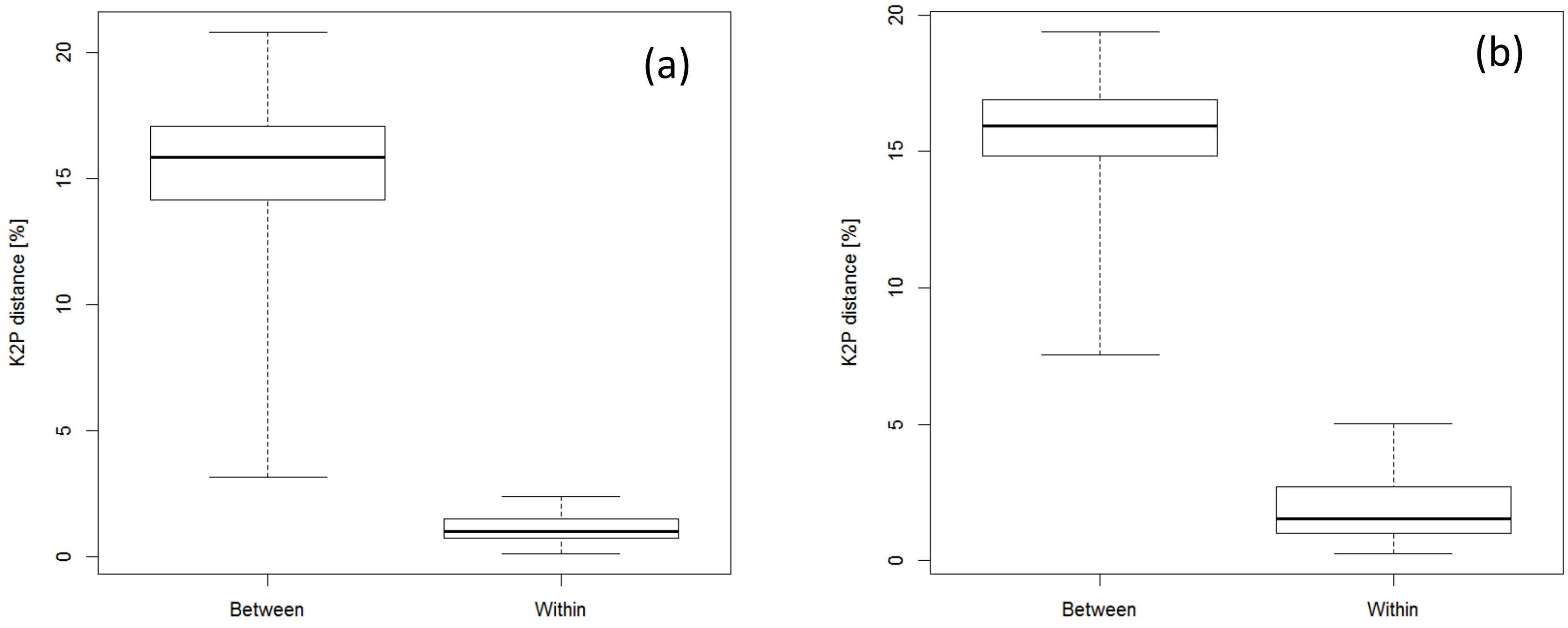

Supplement: Additional file 4: — Distribution of genetic distances at CYTB within and among taxa delimited by different methods. [file 12862_2014_256_MOESM4_ESM.pdf]
