# Supplementary material for: Pan-African phylogeny of Mus (subgenus Nannomys) reveals one of the most successful mammal radiations in Africa
Source: BMC Evol Biol. 2014 Dec 14;14:256. doi: 10.1186/s12862-014-0256-2 (PMC4280006; doi:10.1186/s12862-014-0256-2)

Bryja et al., **Additional file 5**: Biogeographical reconstruction of ancestral distribution of *Nannomys* lineages using maximum likelihood in Lagrange. Three main types of distribution were defined: blue - mountains in Eastern Africa, green – tropical forests of central and western Africa; red – savannah-like habitats surrounding forests and mountains in sub-Saharan Africa. Analysis of changes in distribution pattern in Lagrange allowed two ancestral states in the same time. Pie-charts represent the relative probability that the ancestors of each daughter branch occupied a given region immediately after speciation.

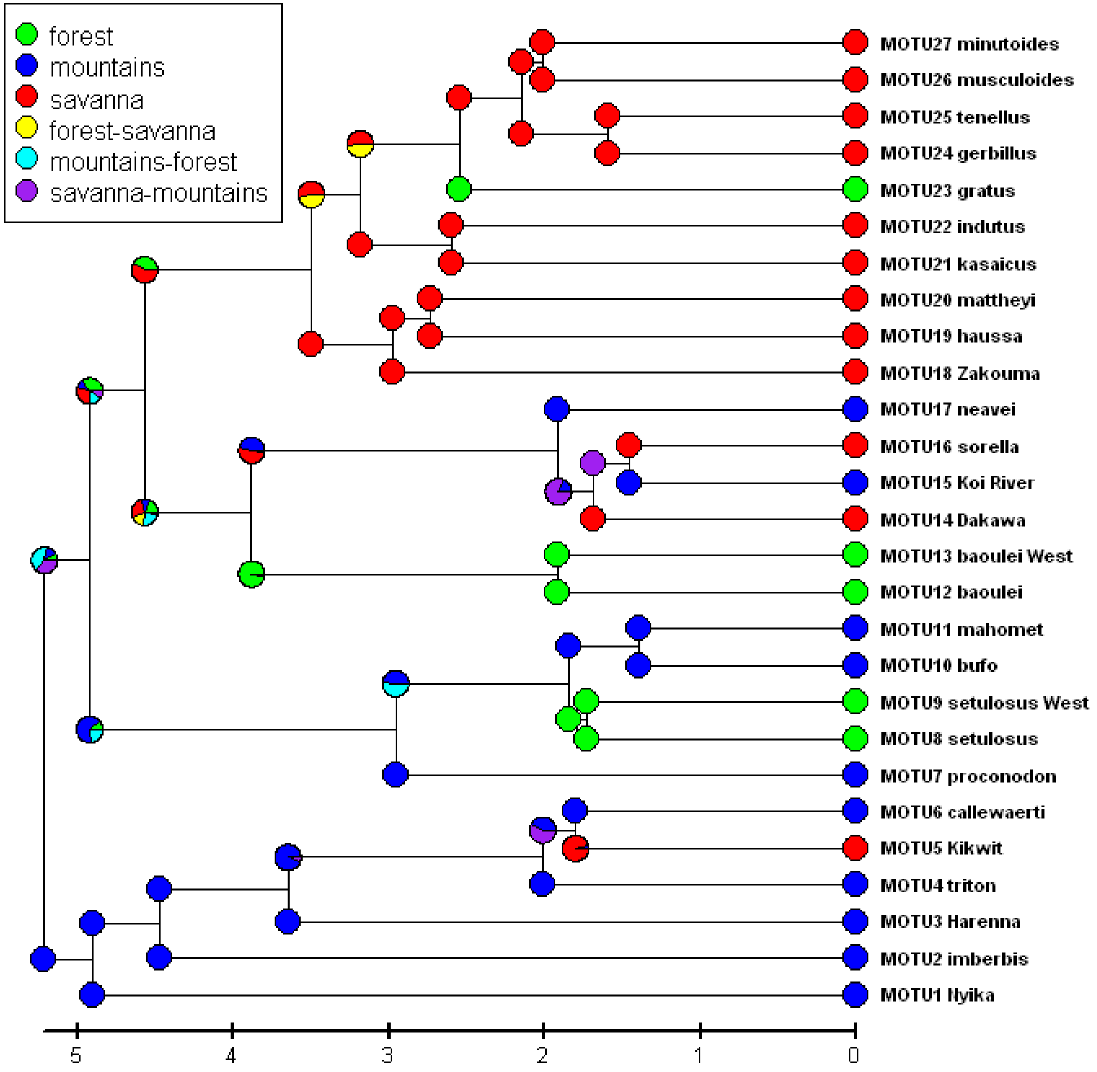

Supplement: Additional file 5: — Biogeographical reconstruction of ancestral distribution of Nannomys lineages using maximum likelihood in Lagrange. [file 12862_2014_256_MOESM5_ESM.pdf]
